# On the effective field theory of heterotic vacua

**DOI:** 10.1007/s11005-017-1025-0

**Published:** 2017-11-13

**Authors:** Jock McOrist

**Affiliations:** Department of Mathematics, University of Surrey, Guildford, GU2 7XH UK

**Keywords:** Superstring theories, Supersymmetric field theories, String and supergravity theories, Kaluza-Klein and other higher-dimensional theories, 81T30, 81T60, 83E15, 83E30, 83E50

## Abstract

The effective field theory of heterotic vacua that realise  preserving $\mathcal {N}{=}1$ supersymmetry is studied. The vacua in question admit large radius limits taking the form , with  a smooth threefold with vanishing first Chern class and a stable holomorphic gauge bundle . In a previous paper we calculated the kinetic terms for moduli, deducing the moduli metric and Kähler potential. In this paper, we compute the remaining couplings in the effective field theory, correct to first order in ${\alpha ^{\backprime }\,}$. In particular, we compute the contribution of the matter sector to the Kähler potential and derive the Yukawa couplings and other quadratic fermionic couplings. From this we write down a Kähler potential  and superpotential .

## Introduction

We are interested in heterotic vacua that realise $\mathcal {N}{=}1$ supersymmetric field theories in . At large radius, these take form  where  is a compact smooth complex threefold with vanishing first Chern class. We study the $E_8{\times } E_8$ heterotic string, and so there is a holomorphic vector bundle  with a structure group  and a $d=4$ spacetime gauge symmetry given by the commutant . The bundle  has a connection *A*, with field strength *F* satisfying the Hermitian Yang–Mills equation. The field strength *F* is related to a gauge-invariant three-form *H* and the curvature of  through anomaly cancellation. The triple  forms a heterotic structure, and the moduli space of these structures is described by what we call heterotic geometry. In this paper, we compute the contribution of fields charged under the spacetime gauge group ${\mathfrak G}$ to the heterotic geometry.

The challenge in studying heterotic vacua is the complicated relationship between *H*, the field strength *F* and the geometry of . Supersymmetry relates the complex structure *J* and Hermitian form $\omega$ of  to the gauge-invariant three-form *H*:

1.1 $$\begin{aligned} H = \text {d}^c \omega , \qquad \text {d}^c\omega = \frac{1}{2}J_{m_1}{}^{n_1}J_{m_2}{}^{n_2} J_{m_3}{}^{n_3} (\partial _{n_1}\omega _{n_2n_3})\, \text {d}x^{m_1} \text {d}x^{m_2} \text {d}x^{m_3}. \end{aligned}$$

where $x^m$ are real coordinates on . Green–Schwarz anomaly cancellation gives a modified Bianchi identity for *H*

1.2 $$\begin{aligned} \text {d}H =- \frac{{\alpha ^{\backprime }\,}}{4} \left( \,\text {Tr}\,F^2 - \,\text {Tr}\,R^2\, \right) , \end{aligned}$$

where in the second of these equations *R* is the curvature two-form computed with respect to a appropriate connection with torsion proportional to *H*. This means the tangent bundle  has torsion if *H* is nonzero. Unless one is considering the standard embedding—in which  is identified with  the tangent bundle to —the right-hand side of (1.2) is nonzero even when  is a Calabi–Yau manifold at large radius. This means that *H* is generically non-vanishing, though subleading in ${\alpha ^{\backprime }\,}$, and so even for large radius heterotic vacua  is non-Kähler. Torsion is inescapable.

The effective field theory of the light fields for these vacua is described by a Lagrangian with $\mathcal {N}=1$ supersymmetry, whose bosonic sector is of the form

1.3 $$\begin{aligned} \mathcal {L}= \frac{1}{2\kappa _4^2} \sqrt{-G_4} \left( \mathcal {R}_4 -\frac{1}{4}\,\text {Tr}\,|F_{\mathfrak {g}}|^2 - 2G_{A {\overline{B}}} \widehat{\mathcal {D}}_e \Phi ^A \widehat{\mathcal {D}}_e \Phi ^{\overline{B}} - V(\Phi ,\bar{\Phi }) + \cdots \right) .\nonumber \\ \end{aligned}$$

Here $\kappa _4$ is the four-dimensional Newton constant, $\mathcal {R}_4$ is the four-dimensional Ricci scalar, $F_{\mathfrak {g}}$ is the spacetime gauge field strength, the $\Phi ^A$ is range over the scalar fields of the field theory, and their kinetic term comes with a metric $G_{A{\overline{B}}}$. The fields $\Phi ^A$ may be charged under ${\mathfrak {g}}$, the algebra of the gauge group ${\mathfrak G}$, with an appropriate covariant derivative $\widehat{\mathcal {D}}_e$. Finally $V(\Phi ,\bar{\Phi })$ is the bosonic potential for the scalars.

When  the moduli space of the heterotic theory reduces to that of a Calabi–Yau manifold and is described by special geometry. The unbroken gauge group in spacetime is $E_6$, and the charged matter content consists of fields charged in the ${{\varvec{27}}}$ and ${{\overline{\varvec{27}}}}$ representations. The Yukawa couplings were calculated in supergravity in, for example, [1, 2]. The effective field theory of this compactification was described in a beautiful paper [3], in which relations between the Kähler potential and superpotential were computed using string scattering amplitudes, (2, 2) supersymmetry and Ward identities. The Kähler and superpotential were shown to be related to each other and in fact were both determined in terms of a pair of holomorphic functions. These are known as the special geometry relations. For a review of special geometry in the language of this paper, see [4]. A key question is how these relations generalise to other choices of bundle .

We work towards answering this question by computing the effective field theory couplings correct to first order in ${\alpha ^{\backprime }\,}$. In a previous paper [5] we commenced a study of heterotic geometry using ${\alpha ^{\backprime }\,}$-corrected supergravity. This is complementary to a series of papers [6–10] who identified the parameter space with certain cohomology groups. In the context of effective field theory (1.3), one of the results of [5] was to calculate the contribution of the bosonic moduli fields to the metric $G_{A{\overline{B}}}$. In this paper, we compute the contribution of the matter sector to the metric $G_{A{\overline{B}}}$, and the Yukawa couplings, correct to order ${\alpha ^{\backprime }\,}$. We describe an ansatz for the superpotential and Kähler potential for effective field theory:1.4The superpotential is normalised by comparing with the Yukawa couplings computed in the dimensional reduction using the conventions of Wess–Bagger [31].

The moduli have a metric

1.5 $$\begin{aligned} \begin{aligned} \text {d}s^2&= 2G_{\alpha {\overline{\beta }}} \,\text {d}y^\alpha \otimes \text {d}y^{\overline{\beta }}, \\ G_{\alpha {\overline{\beta }}}&= \frac{1}{4V} \int \Delta _\alpha {}^\mu \star \Delta _{\overline{\beta }}{}^\nu \,\, g_{\mu {\bar{\nu }}} + \frac{1}{4V} \int \mathcal {Z}_\alpha \star \mathcal {Z}_{\overline{\beta }}\,+ \\&\qquad + \frac{ {\alpha ^{\backprime }\,}}{4V}\int \,\text {Tr}\,\Big ( D_\alpha A \star D_{\overline{\beta }}A \Big ) - \frac{ {\alpha ^{\backprime }\,}}{4V}\int \,\text {Tr}\,\Big (D_\alpha \Theta \,\star \, D_{\overline{\beta }}\Theta ^\dag \Big ), \end{aligned} \end{aligned}$$

where  is the ${\alpha ^{\backprime }\,}$-corrected, gauge-invariant generalisation of the complexified Kahler form $\delta B + \text {i}\delta \omega$, the $\chi _\alpha$ form a basis of closed (2, 1)-forms, and the last line is the Kobayashi metric, extended to the entire parameter space, including deformations of the spin connection on . The metric expressed this way is an inner product of tensors corresponding to complex structure $\Delta _\alpha$, Hermitian moduli , and bundle moduli $D_\alpha A$. The role of the spin connection $D_\alpha \theta$ is presumably determined in terms of the other moduli as they do not correspond to independent physical fields. The tensors depend on parameters holomorphically through1.6de la Ossa and Svanes [6] showed that there exists a choice of basis for the parameters in which each of the tensors in the metric are in an appropriate cohomology;^1^ hence, the moduli space metric (5.2) is the natural inner product (Weil–Peterson) on cohomology classes.

The matter fields are $C^\xi$ and  and appear in the Kähler potential trivially, as they do in special geometry. The matter metric is the Weil–Petersson inner product of corresponding cohomology elements1.7where $\phi _\xi , \psi _\rho$ are (0, 1)-forms valued in a sum over representations of the structure group $\mathcal {H}$.

In some sense it was remarkable that one was able to find a compact closed expression for the Kähler potential for the moduli metric. This was not a priori obvious, especially given the nonlinear PDEs relating parameters in the anomalous Bianchi identity and supersymmetry relations (1.1)–(1.2). Indeed, it turned out that the Kähler potential for the moduli in (1.4) is of the same in form as that of special geometry, except where one has replaced the Kähler form by the Hermitian form $\omega$. At first sight this is confusing as the only fields appearing in the Kähler potential are $\omega$ and $\Omega$. Nonetheless, the Kähler potential still depends on bundle moduli in precisely the right way through a non-trivial analysis of the supersymmetry and anomaly conditions. The Hermitian form $\omega$ contains, hidden within, information about both the bundle and Hermitian moduli.^2^

The metric (5.2) is compatible with the result in [11], who studied the ${\alpha ^{\backprime }\,}$ and ${\alpha ^{\backprime }\,}^2$ corrections to the moduli space metric in the particular case where the Hermitian part of the metric varies, while the remaining fields are fixed: $(\partial _a \omega )^{1,1} \ne 0$, . In general all fields vary with parameters and the metric is nonzero already at $\mathcal {O}({\alpha ^{\backprime }\,})$.

The analysis in [5] focussed primarily on D-terms relevant to moduli. In this paper, we compute the remaining D-terms, including the metric terms for the bosonic matter fields charged under ${\mathfrak {g}}$. We also compute the F-terms up to cubic order in fields, exploiting the formalism constructed in [5]. The primary utility of this is to derive an expression for the Yukawa couplings in a manifestly covariant fashion. Together with the metrics discussed above, one is now finally able to compute properly normalised Yukawa couplings, relevant to any serious particle phenomenology. The F-terms are protected in ${\alpha ^{\backprime }\,}$-perturbation theory, and so the only possible ${\alpha ^{\backprime }\,}$-corrections are due to worldsheet instantons.

The fields neutral under ${\mathfrak {g}}$, the singlet fields, also do not have any mass or cubic Yukawa couplings. In fact, all singlet couplings necessarily vanish. They correspond to moduli which are necessarily free parameters and so the singlets need to have unconstrained vacuum expectation values. If there were a nonzero singlet coupling at some order in the field expansion, e.g. ${{\varvec{1}}}^n$, or in a ${\mathfrak {e}}_6$ theory $({{\varvec{27}}}\cdot {{\overline{\varvec{27}}}})^{326}\cdot {{\varvec{1}}}^{101}$, then some parameter $y^\alpha$ would have its value fixed, a contradiction on it being a free parameter.^3^

The superpotential  in (1.4) is an ansatz designed to replicate these couplings. Its functional form can be partly argued by symmetry. There is a complex line bundle over the moduli space in which the holomorphic volume form on , denoted $\Omega$, transforms with a gauge symmetry $\Omega \rightarrow \mu \Omega$ where . The superpotential is also a section of this line bundle, and transforms in the same way . Hence,  has an integrand proportional to $\Omega$. To make the integrand a nice top-form we need to wedge it with a gauge-invariant three-form. The three-form needs to contain a dependence on the matter fields, and this can only occur through the ten-dimensional *H* field. The other natural gauge-invariant three-forms that are not defined in a given complex structure are $\text {d}\omega$ and $\text {d}^c\omega$.  is also required not to give rise to any singlet couplings. So all derivatives of  with respect to parameters must vanish. The combination $H - \text {d}^c \omega$ manifestly satisfies this request. Derivatives with respect to matter fields of  do not vanish. As these are charged in ${\mathfrak {g}}$, the only nonzero contributions come from *H*. This allows us to fix the normalisation of  by comparing with the dimensional reduction calculation of the Yukawa couplings. Finally,  must be a holomorphic function of chiral fields, which is straightforward to check. It is convenient that the single expression for the superpotential captures both the matter and moduli couplings, and fact seemingly not realised before.

A complementary perspective on  was studied by [8]. In that paper, one starts with an ${\mathfrak {su}}(3)$-structure manifold , posits the existence of , and uses it as a device to reproduce the conditions needed for the heterotic vacuum to be supersymmetry. This builds on earlier work in the literature, see, for example, [14–16]. The superpotential ansatzed in those papers is of a different form to that described here, and the cubic and higher-order singlet couplings nor Yukawa couplings were not consistently computed. We choose to work with the expression above as it manifestly replicates the vanishing of all singlet couplings.

The layout of this paper is the following. In Sect. 2 we review the necessary background to study heterotic vacua, reviewing the results of [5]. In Sect. 3, we dimensionally reduce the Yang–Mills sector to obtain a metric on the matter fields. In Sect. 4, the reduction is applied to the gaugino to get the quadratic fermionic couplings, including the Yukawa couplings. In Sect. 5, we summarise the results. In Sect. 6 we show how these couplings are represented in the language of a Kähler potential  and superpotential .

### Tables of notation

**Table 1:** A table of objects used

Quantity	Definition	Comment
	$d=4$ spacetime gauge algebra	Group is ${\mathfrak G}$
${\mathfrak {h}}$	Structure algebra of	Group is
${{\varvec{r}}}$	Representation of ${\mathfrak {h}}$	$\dim {{\varvec{r}}}= r$
${{\varvec{R}}}$	Representation of ${\mathfrak {g}}$	$\dim {{\varvec{R}}} = R$
$\Phi$	$d=10$ gauge field in $({{\varvec{r}}},{{\overline{\varvec{R}}}})$ of ${\mathfrak {h}}\oplus {\mathfrak {g}}$	$\phi = \Phi ^{0,1}$, $\Phi = \phi - \psi ^\dag$
$\Psi$	$d=10$ gauge field in $({{\overline{\varvec{r}}}},{{\varvec{R}}})$ of ${\mathfrak {h}}\oplus {\mathfrak {g}}$	$\psi = \Psi ^{0,1}$, $\Psi = \psi - \phi ^\dag$
$\phi _{\xi }$	Basis for	Valued in ${{\varvec{r}}}$ of ${\mathfrak {h}}$
$\psi _{\rho }$	Basis for	Valued in ${{\overline{\varvec{r}}}}$ of ${\mathfrak {h}}$
$C^{\xi }, D^{\rho }, Y^\alpha$	$d=4$ bosons in the ${{\overline{\varvec{R}}}}$, ${{\varvec{R}}}$, ${{\varvec{1}}}$ of ${\mathfrak {g}}$ (e.g. ${{\overline{\varvec{27}}}}$, ${{\varvec{27}}}$, ${{\varvec{1}}}$ of ${\mathfrak {e}}_6$)	$\xi ,\tau ,\alpha$ label harmonic bases
$\mathcal {C}^\xi , \mathcal {D}^\rho , \mathcal {Y}^\alpha$	$d=4$ fermions in ${{\overline{\varvec{R}}}}$, ${{\varvec{R}}}$ of ${\mathfrak {g}}$	Calligraphic for anticommuting
$B_e \,\text {d}X^e$	${\mathfrak {g}}$-valued connection on	Occasionally embed in $A_{{\mathfrak {e}}_8}$
$A_m\, \text {d}x^m$	${\mathfrak {h}}$-valued connection for on	Occasionally embed in $A_{{\mathfrak {e}}_8}$
$\delta \mathcal {A}$	Fluctuation of connection for	Occasionally $\delta A_{{\mathfrak {h}}}$
$\delta B$	Fluctuation of connection for ${\mathfrak {g}}$	Occasionally use $\delta A_{{\mathfrak {g}}}$
$\varepsilon$	Majorana–Weyl ${\mathfrak {so}}(9,1)$ spinor	
$\zeta \otimes \lambda$	${\mathfrak {so}}(3,1)\oplus {\mathfrak {so}}(6)$ spinors	$\lambda , \lambda '$ positive/negative chirality

**Table 2:** A table of coordinates and indices

Coordinates		Holomorphic indices	Real indices
Calabi–Yau manifold	$x^\mu$	$\mu ,\,\nu ,\ldots$	$m,n,\ldots$
spacetime	$X^e$	−	$e,\,f,\ldots$
basis for rep ${{\varvec{r}}}$ of ${\mathfrak {h}}$ e.g. ${\mathfrak {h}}= {\mathfrak {su}}(3)$	$[T_{\mathfrak {h}}]^i{}_j \in {{\varvec{r}}}$	$i,j=1,\ldots , {r}$	−
basis for rep ${{\varvec{R}}}$ of ${\mathfrak {g}}$ e.g. ${\mathfrak {g}}= {\mathfrak {e}}_6$	$[T_{\mathfrak {g}}]^{M}{}_N \in {{\varvec{R}}}$	$M,N=1,\ldots ,{R}$	−
parameters of heterotic structure	$y^\alpha$	$\alpha ,\,\beta , \gamma ,\ldots$	$a,\,b,c\ldots$
indices for $d=4$ spinors (occasional)	$\zeta _a, {\overline{\zeta }}^{\dot{a}}$	−	*a*, *b*,

## Heterotic geometry

The purpose of this section is to establish conventions and notation through a review of heterotic moduli geometry, most of which is explained in [5]. In terms of notation, there are occasional refinements and new results towards the end of the section. We largely work in the notation of [5], with a few exceptions, most important of which is that real parameters are denoted by $y^a$ and complex parameters by $y^\alpha , y^{\overline{\beta }}$. The discussion both there and in this section refers to forms defined on the manifold . This is generalised in later sections in order to account for the charged matter fields. A table of notation is given in Tables 1 and 2. Basic results and a summary of conventions are found in Appendices. Hodge theory and forms are in “Appendix A”; spinors in “Appendix B”; and representation theory in “Appendix C”.

We consider a geometry  with  smooth, compact, complex and vanishing first Chern class. While  is not Kähler in general, we take it to be cohomologically Kähler satisfying the $\partial \overline{\partial }$-lemma, meaning that its cohomology groups are that of a Calabi–Yau manifold.

The heterotic action is fixed by supersymmetry up to and including $\alpha '^2$–corrections. In string frame with an appropriate choice of connection for , it takes the form [17, 18]:

2.1 $$\begin{aligned} S = \frac{1}{2\kappa _{10}^2} \int \! \text {d}^{10\,}\! X \sqrt{g_{10}}\, e^{-2\Phi } \Big \{ \mathcal {R}- \frac{1}{2}|H|^2 + 4(\partial \Phi )^2 - \frac{\alpha '}{4}\big ( \,\text {Tr}\,|F|^2 {-} \,\text {Tr}\,|R(\Theta ^+)|^2 \big ) \Big \}, \end{aligned}$$

Our notation is such that $\mu ,\nu ,\ldots$ are holomorphic indices along  with coordinates *x*; $m,n,\ldots$ are real indices along ; while $e,f,\ldots$ are spacetime indices corresponding to spacetime coordinates . The 10-dimensional Newton constant is denoted by $\kappa _{10}$, $g_{10}=-\det (g_{MN})$, $\Phi$ is the 10-dimensional dilaton, $\mathcal {R}$ is the Ricci scalar evaluated using the Levi–Civita connection and *F* is the Yang–Mills field strength with the trace taken in the adjoint of the gauge group.

We define an inner product on *p*-forms by

$$\begin{aligned} \langle S,\, T\rangle = \frac{1}{p!} \, g^{M_1 N_1} \ldots g^{M_p N_p}\, S_{M_1\ldots M_p} \,T_{N_1 \ldots N_p}. \end{aligned}$$

and take the *p*-form norm as

$$\begin{aligned} |T|^2 =\langle T,\, T\rangle . \end{aligned}$$

Thus, the curvature squared terms correspond to

$$\begin{aligned} \,\text {Tr}\,|F|^2 = \frac{1}{2}\,\text {Tr}\,F_{MN} F^{MN}~~~\text {and}~~~ \,\text {Tr}\,|R(\Theta ^+)|^2 = \frac{1}{2}\,\text {Tr}\,R_{MNPQ}(\Theta ^+) R^{MNPQ}(\Theta ^+), \end{aligned}$$

where the Riemann curvature is evaluated using a twisted connection

$$\begin{aligned} \Theta ^\pm _M = \Theta _M \pm \frac{1}{2}H_M, \end{aligned}$$

with $\Theta _M$ is the Levi–Civita connection. The definition of the *H* field strength and its gauge transformations are given in Sect. 2.3.

We write the metric on  as

$$\begin{aligned} \text {d}s^2 = 2g_{\mu {\bar{\nu }}} \text {d}x^\mu \text {d}x^{\bar{\nu }}. \end{aligned}$$

The manifold  has a holomorphic (3, 0)-form

$$\begin{aligned} \Omega = \frac{1}{3!} \Omega _{\mu \nu \rho } \text {d}x^\mu \text {d}x^\nu \text {d}x^\rho , \end{aligned}$$

where $\Omega _{\mu \nu \rho }$ depends holomorphically on parameters and coordinates of . $\Omega$ is a section of a line bundle over the moduli space, meaning that there is a gauge symmetry in which $\Omega \rightarrow \mu \Omega$ where  is a holomorphic function of parameters.

There is a compatibility relation

2.2 $$\begin{aligned} \frac{\text {i}}{ ||\Omega ||^2} \Omega \, {\overline{ \Omega }}= \frac{1}{3!}\omega ^3, \qquad ||\Omega ||^2 = \frac{1}{3!} \Omega _{\mu \nu \rho } {\overline{ \Omega }}^{\mu \nu \rho }, \end{aligned}$$

where ${||\Omega ||}$ is the norm of $\Omega$. For fixed , this is often normalised so that $||\Omega ||^2 =8$. However, ${||\Omega ||}$ depends on moduli and is gauge dependant and so it is not consistent in moduli problems to do this.

### Derivatives of $\Omega$ and $\Delta _\alpha$

A variation of complex structure given by a parameter $y^\alpha$ can be described in terms of the variation of the holomorphic three-form by noting that $\partial _\alpha \Omega \in H^{(3,0)}\oplus H^{(2,1)}$ and writing

2.3 $$\begin{aligned} \frac{\partial \Omega }{\partial y^\alpha }=-\,k_\alpha \,\Omega + \chi _\alpha ~;~~~ \chi _\alpha =\frac{1}{2} \chi _{\alpha \,\kappa \lambda \bar{\nu }}\,\text {d}x^\kappa \text {d}x^\lambda \text {d}x^{\bar{\nu }}. \end{aligned}$$

Here $\chi _\alpha$ are $\overline{\partial }$-closed (2, 1)-forms. Variations of complex structure  can also be phrased in terms of (0, 1)-forms valued in

$$\begin{aligned} \partial _\alpha J = 2\text {i}\,\Delta _{\alpha \,{\bar{\nu }}}{}^\mu \text {d}x^{\bar{\nu }}\otimes \partial _\mu , \qquad \partial _\alpha \partial _\beta J = 2\text {i}\,\Delta _{\alpha \beta } = 2\text {i}\,\Delta _{\alpha \beta }{}_{\bar{\nu }}{}^\mu \text {d}x^{\bar{\nu }}\otimes \partial _\mu . \end{aligned}$$

We have denoted $\partial _\beta \Delta _\alpha = \Delta _{\alpha \beta }$ which makes manifest the symmetry property $\partial _\beta \Delta _\alpha = \partial _\alpha \Delta _\beta$. Occasionally we will denote parameter derivatives by .

The $\Delta _\alpha$ and $\chi _\alpha$ are related2.4The symmetric component of $\Delta _\alpha {}^\mu$ appears in variations of the metric $\delta g_{\bar{\mu }\bar{\nu }}=\Delta _{\alpha \,(\bar{\mu }\bar{\nu })}\, \delta y^\alpha$.

It is best to describe variations of complex structure through projectors *P* and *Q* onto holomorphic and antiholomorphic components, respectively,

$$\begin{aligned} P_m{}^n = \frac{1}{2}\left( \delta _m{}^n - \text {i}J_m{}^n\right) , \qquad Q_m{}^n = \frac{1}{2}\left( \delta _m{}^n + \text {i}J_m{}^n\right) . \end{aligned}$$

The projectors capture the implicit dependence on complex structure. For example, the operator $\overline{\partial }=\text {d}x^m Q_m{}^n\, \partial _n$ undergoes a variation purely as a consequence of the implicit dependence on the complex structure:

2.5 $$\begin{aligned}{}[\partial _\alpha ,\overline{\partial }] =\left[ \partial _\alpha , (Q_m{}^n \text {d}x^m \otimes \partial _n)\right] = - \Delta _\alpha {}^\mu \partial _\mu \end{aligned}$$

### The vector bundle 

Let  denote a vector bundle over , with structure group $\mathcal {H}$, and *A* the connection on the associated principal bundle. That is, *A* is a gauge field valued in the adjoint representation $\mathrm{ad }_{\mathfrak {h}}$ of the Lie algebra ${\mathfrak {h}}$ of $\mathcal {H}$.

Under a gauge transformation, *A* has the transformation rule

2.6 $$\begin{aligned} ^{\Phi }A = \Phi (A - Y)\Phi ^{-1}, \quad Y=\Phi ^{-1}\text {d}\Phi , \end{aligned}$$

where $\Phi$ is a function on  that takes values in ${\mathfrak G}$. We take $\Phi$ to be unitary and then $\text {d}\Phi \,\Phi ^{-1}$ and *A* are anti-Hermitian. The field strength is

$$\begin{aligned} F=\text {d}A + A^2, \end{aligned}$$

and this transforms in the adjoint of the gauge group: $F\rightarrow \Phi F\Phi ^{-1}$.

Let $\mathcal {A}$ be the (0, 1) part of *A* then, since *A* is anti-Hermitian,

$$\begin{aligned} A = \mathcal {A}- \mathcal {A}^{\dag }. \end{aligned}$$

On decomposing the field strength into type, we find $F^{0,2} = \overline{\partial }\mathcal {A}+ \mathcal {A}^2$. The bundle  is holomorphic if and only if there exists a connection such that $F^{(0,2)}=0$. The Hermitian Yang–Mills equation is

$$\begin{aligned} \omega ^2 F =0. \end{aligned}$$

### The *B* and *H* fields

There is a gauge-invariant three-form

2.7 $$\begin{aligned} H=\text {d}B - \frac{{\alpha ^{\backprime }\,}}{4}\Big ({\text {CS}}[A] - {\text {CS}}[\Theta ]\Big ), \end{aligned}$$

where ${\text {CS}}$ denotes the Chern-Simons three-formand $\Theta$ is the connection on  for Lorentz symmetries. The three-form $\text {d}B$ is defined so that *H* to be gauge invariant, and so $\text {d}B$ itself has gauge transformations. Under a gauge transformation

$$\begin{aligned} {\text {CS}}[A]~\rightarrow ~{\text {CS}}[A] - \text {d}\,\text {Tr}\,\!(AY) + \frac{1}{3} \,\text {Tr}\,\left( Y^3\right) , \end{aligned}$$

together with the analogous rule for ${\text {CS}}[\Theta ]$. The integral of $\,\text {Tr}\,(Y^3)$ over a three-cycle is a winding number, so it vanishes if the gauge transformation is continuously connected to the identity. The integral vanishes for every three-cycle and so $\,\text {Tr}\,(Y^3)$ is exact $\frac{1}{3}\,\,\text {Tr}\,(Y^3) =\text {d}U,$ for some globally defined two-form *U*. There are corresponding transformations for the connection $\Theta$ in which *Y* is replaced by *Z* and *U* by *W*.

Anomaly cancellation condition means that the *B* field is assigned a transformation

2.8 $$\begin{aligned} B~\rightarrow ~B - \frac{{\alpha ^{\backprime }\,}}{4}\left\{ \,\text {Tr}\,(AY) - U - \,\text {Tr}\,(\Theta Z) + W\right\} . \end{aligned}$$

With this transformation law, *B* is a 2-gerbe and *H* is invariant.

An important constraint arising from supersymmetry is that *H* is related to the Hermitian form $\omega$ and complex structure *J* of :

2.9 $$\begin{aligned} H = \text {d}^c\omega , \qquad \text {d}^c \omega = \frac{1}{2}J_{m_1}{}^{n_1}J_{m_2}{}^{n_2} J_{m_3}{}^{n_3} (\partial _{n_1}\omega _{n_2n_3})\, \text {d}x^{m_1} \text {d}x^{m_2} \text {d}x^{m_3}, \end{aligned}$$

which for an integrable complex structure reduces to

2.10 $$\begin{aligned} \text {d}^c \omega = J^m \partial _m \omega - (\text {d}J^m) \omega _m. \end{aligned}$$

We denote the real parameters of the compactification by $y^a$ and complex parameters by $y^\alpha ,y^{\overline{\beta }}$. If the parameters are fixed to $y=y_0$, the second term in (2.10) vanishes and the relation simplifies to

2.11 $$\begin{aligned} \text {d}^c \omega |_{y=y_0} = \text {i}(\partial - \overline{\partial }) \omega . \end{aligned}$$

However, when complex structure is varied $\partial _\alpha (\text {d}J) = 2\text {i}\partial \Delta _\alpha$, the second term in (2.10) is nonzero and is important as it contributes to the equations satisfied by the moduli.

### Derivatives of *A*

The heterotic structure  depends on parameters. This means the gauge connection *A* and its gauge transformations $\Phi$ depend on parameters. As constructed in [5], the gauge covariant way of describing a deformation of *A* is given by introducing a covariant derivative

2.12 $$\begin{aligned} D_a A=\partial _a A - \text {d}_A A_a, \end{aligned}$$

where $A^\sharp = A_a \text {d}y^a$ is a connection on the moduli space with a transformation law

2.13 $$\begin{aligned} {}^\Phi A_a =\Phi (A_a - Y_a)\Phi ^{-1},\qquad Y_a = \Phi ^{-1}\,\partial _a \Phi . \end{aligned}$$

With this transformation property $D_a A$ transforms homogeneously under (2.6):

$$\begin{aligned} D_a A~\rightarrow ~ \Phi \,D_a A\,\Phi ^{-1}. \end{aligned}$$

The moduli space $\mathcal {M}$ is complex, and we introduce a complex structure $y^a = (y^\alpha , y^{\overline{\beta }})$. When parameters vary complex structure the holomorphic type of forms change, and the covariant derivatives $D_\alpha \mathcal {A}$ is no longer gauge covariant. This is remedied by defining a generalisation, termed the holotypical derivative :2.14where the vanishing of  follows from (2.5). It follows from the definition that under a gauge transformation the holotypical derivative transforms in the desired formWithout the extra term $- \Delta _\alpha {}^\mu \mathcal {A}^\dag _\mu$ in the holotypical derivative, this property does not hold as $\overline{\partial }$ fails to commute with $\partial _\alpha$.

The holotypical derivative can be extended to act on (*p*, *q*)-forms. Define

$$\begin{aligned} W_m^{r,s} = \frac{1}{r!s!}\, W_{m \mu _1\cdots \mu _r {\bar{\nu }}_1\cdots {\bar{\nu }}_s} \text {d}x^{\mu _1\cdots \mu _r{\bar{\nu }}_1\cdots {\bar{\nu }}_s}, \end{aligned}$$

and understand $W_m^{r,s} = 0$ if *r* or *s* are negative or $r{+}s>n{-}1$. The holotypical derivatives are then given by2.15The holotypical derivative has the nice feature that it preserves holomorphic type:We use $D_\alpha$ to denote the covariant derivative to account for any gauge dependence of the real form *W*. For example, the covariant derivative of the field strength is related to that of *A*:

$$\begin{aligned} D_M F=\partial _M F + \left[ A^\sharp _M, F\right] = \text {d}_A (D_M A), \end{aligned}$$

However, the holotypical derivative of, for example, $F^{(0,2)}$ givesand this is known as the Atiyah constraint.

### Derivatives of *H*

It is of use to compute derivatives of *H* with respect to parameters. First define a gauge covariant derivative of *B* via

2.16 $$\begin{aligned} D_a B = \partial _a B -\frac{{\alpha ^{\backprime }\,}}{4} \,\text {Tr}\,( A_a\,\text {d}A), \end{aligned}$$

With this choice, we have a gauge transformation law for $D_a B$ that is parallel to the gauge transformation (2.8) for *B*:

2.17 $$\begin{aligned} {}^\Phi D_a B= D_a B + \frac{{\alpha ^{\backprime }\,}}{4}\Big (\! \,\text {Tr}\,( Y D_a A ) + U_a \Big ). \end{aligned}$$

The second and third derivatives are defined to transform in a natural way inherited from that of $D_a B$

2.18 $$\begin{aligned} \begin{aligned} D_a D_b B&= \partial _a D_b B - \frac{{\alpha ^{\backprime }\,}}{4} \,\text {Tr}\,( D_b A \text {d}A_a),\\D_cD_a D_b B&= \partial _c D_a D_b B - \frac{{\alpha ^{\backprime }\,}}{4} \,\text {Tr}\,\left( D_b D_a A\, \text {d}A^\sharp _c\right) . \end{aligned} \end{aligned}$$

A gauge-invariant quantity  is the formed from $D_a B$
2.19with $\text {d}b_a$ an exact form. The exact form comes from the fact the physical quantity is $\text {d}B$, and so in writing  there is a corresponding ambiguity. It is a simple exercise to note that $\partial _a H$ is given by the expression2.20In terms of holomorphic parameters, we introduce the holotypical derivative and find2.21The second derivative is given by2.22where  and in terms of the B-fieldDespite appearances the right-hand side is symmetric in *a*, *b* after one uses that2.23and so2.24The third derivative of *H* is given by2.25where  and in terms of the B-field:For similar reasons to the second derivative above, this is actually symmetric in *a*, *b*, *c*, but not made manifest in this expression for compactness.

### Derivatives of $\text {d}^c \omega$

The derivative of $\text {d}^c \omega$ in (2.10) with respect to parameters is2.26We can evaluate (2.26) for a given complex structure, denoted $|_{y_0}$ in the corresponding complex coordinates of :For a given complex structure, we project onto holomorphic type then we need to use holotypical derivatives. Two cases we will need and then projecting onto (0, 3) and (1, 2) components2.27The second derivative of $\text {d}^c\omega$, given by differentiating (2.26) and then evaluated on a fixed complex structure, is

$$\begin{aligned} \begin{aligned} \left( \partial _\alpha \partial _\beta \text {d}^c\omega \right) |_{y_0}&=~ 2\text {i}\left( \Delta _{\alpha \beta }{}^\mu \right) (\partial _\mu \omega ) + 2\text {i}\Delta _\alpha {}^\mu \partial _\mu (\partial _\beta \omega ) \\&\quad +\,2\text {i}\Delta _\beta {}^\mu \partial _\mu (\partial _\alpha \omega ) - 2\text {i}\,\left( \partial \Delta _{\alpha \beta }{}^\mu \right) \omega _\mu \\&\quad +\,\text {i}(\partial -\overline{\partial }) \omega _{,\alpha \beta } -2\text {i}\left( \partial \Delta _\alpha {}^\mu \right) \omega _{\mu ,\beta } - 2\text {i}\left( \partial \Delta _\beta {}^\mu \right) \omega _{\mu ,\alpha }. \end{aligned} \end{aligned}$$

We will have need for the (0, 3)-component:

2.28 $$\begin{aligned} \begin{aligned} \Big ( \partial _\alpha \partial _\beta \text {d}^c\omega \Big )|_{y_0}^{0,3}&= 2\text {i}( \Delta _\alpha {}^\mu \Delta _\beta {}^\nu + \Delta _\beta {}^\mu \Delta _\alpha {}^\nu )\partial _\mu \omega _\nu ^{0,1} -\text {i}\overline{\partial }( \omega _{,\alpha \beta })^{0,2}. \end{aligned} \end{aligned}$$

### Supersymmetry relations

One can apply these results to compute how the supersymmetry condition $\text {d}^c \omega = H$ relates the variations of fields. The parameter space coordinates are corrected at order ${\alpha ^{\backprime }\,}$. Differentiating with respect to these corrected coordinates, Eq. (2.9) gives rise to relations between first-order deformations of fields:2.29where $\gamma _\alpha ^{1,1}$ is $\text {d}$-closed (1, 1)-form, and $k_\alpha ^{0,1}$ and $l_\alpha ^{0,1}$ are some (0, 1)-forms. As discussed in [5] in ${\alpha ^{\backprime }\,}$-perturbation theory,  when appropriately gauge fixed.

The heterotic structure are holomorphic functions of parameters. This can be compactly stated as2.30


## The matter field metric

In this section we dimensionally reduce the Yang–Mills term in (2.1) to obtain the metric for the matter fields. Our task divides into two steps. First, determine how $A_{{\mathfrak {e}}_8}$ decomposes under ${\mathfrak {e}}_8\oplus {\mathfrak {e}}_8\supset {\mathfrak {g}}\oplus {\mathfrak {h}}$, using this to form a KK ansatz. For simplicity we will suppress writing the second ${\mathfrak {e}}_8$ sector. Second, use this to dimensionally reduce the $d=10$ action thereby getting an effective field theory metric and Yukawa couplings for the matter fields and construct the Kähler potential.

### Decomposing *A* under ${\mathfrak {g}}\oplus {\mathfrak {h}}\subset {\mathfrak {e}}_8$

The branching rule for ${\mathfrak {g}}\oplus {\mathfrak {h}}\subset {\mathfrak {e}}_8$ is

3.1 $$\begin{aligned} {\mathrm{ad}}_{{\mathfrak {e}}_8} = ({{\varvec{1}}}, {\mathrm{ad}}_{{\mathfrak {h}}}) \oplus ({\mathrm{ad}}_{{\mathfrak {g}}}, {{\varvec{1}}}) \oplus _i ({{\overline{\varvec{R_i}}}}, {{\varvec{r_i}}}) \oplus ({{\varvec{R_i}}}, {{\overline{\varvec{r_i}}}}). \end{aligned}$$

where $\mathrm{ad }$ denotes the relevant adjoint representation. The matter fields transform in representations of ${\mathfrak {g}}\oplus {\mathfrak {h}}$. We denote these representations by ${{\varvec{r_i}}}$ for ${\mathfrak {h}}$ and ${{\varvec{R_i}}}$ of ${\mathfrak {g}}$, respectively. Denote dimensions in the obvious way $\dim {{\varvec{r_i}}} = r_i$ and $\dim {{\varvec{R_i}}} = R_i$. We have allowed for a sum over all the relevant representations ${{\varvec{r_i}}}$ and ${{\varvec{R_i}}}$, including, for example, pseudo-real representations. For simplicity we will often suppress the sum and write a single matter field representations of ${\mathfrak {g}}\oplus {\mathfrak {h}}$ and its conjugate. The generalisation is obvious.

The matrix presentation of the adjoint of ${\mathfrak {e}}_8$ is complicated. For the moment let us suppose we can write the generator in simplified form as

3.2 $$\begin{aligned} T_{{\mathfrak {e}}_8} = \begin{pmatrix} T_{{\mathfrak {h}}} &{} \quad 0\\ 0 &{} \quad T_{{\mathfrak {g}}} \end{pmatrix}. \end{aligned}$$

We do this as a toy model to illustrate the key points of the calculation, and at the end of the day our results will not depend on this presentation.

The background gauge field is

$$\begin{aligned} A_{{\mathfrak {e}}_8} = A_{\mathfrak {h}}\oplus B_{\mathfrak {g}}, \end{aligned}$$

where $A_{\mathfrak {h}}= A_m(x) \text {d}x^m$ is the ${\mathfrak {h}}$ gauge field and we take it to have legs purely along the CYM; $B_{\mathfrak {g}}= B_e(X) \text {d}X^e$ is the  spacetime gauge field valued in ${\mathfrak {g}}$. When there is no ambiguity we will drop the ${\mathfrak {g}}, {\mathfrak {h}}$ subscripts. We indicate this combined Lorentz and gauge structure schematically in matrix notation

$$\begin{aligned} A_{{\mathfrak {e}}_8} = \begin{pmatrix} A_{{\mathfrak {h}}} &{} \quad 0 \\ 0 &{} \quad B_{{\mathfrak {g}}} \\ \end{pmatrix} = A_m \text {d}x^m + B_e \text {d}X^e. \end{aligned}$$

Note that having off-diagonal terms turned on in the background would amount to Higgsing gauge group, which we do not want for the discussion in this paper.

A fluctuation of $A_{{\mathfrak {e}}_8}$ is of the form

3.3 $$\begin{aligned} \begin{aligned} \delta A_{{\mathfrak {e}}_8}&= \delta A_{(\mathrm{ad }_{\mathfrak {h}},{{\varvec{1}}})} \oplus _i \delta A_{({{\varvec{r_i}}},{{\overline{\varvec{R_j}}}})}\oplus _j \delta A_{({{\overline{\varvec{r_j}}}},{{\varvec{R_j}}})} \oplus \delta A_{({{\varvec{1}}}, \mathrm{ad }_{\mathfrak {g}})} \\&=~ \begin{pmatrix} \delta A_{{\mathfrak {h}}} &{} \quad \Phi \\ \Psi &{} \quad \delta B_{{\mathfrak {g}}} \\ \end{pmatrix}, \qquad \Phi _{r\times R} = \begin{pmatrix} \Phi ^1&\cdots&\Phi ^R \end{pmatrix}, \qquad \Psi _{R\times r} = \begin{pmatrix} \Psi ^1 \\ \vdots \\ \Psi ^R \end{pmatrix}, \end{aligned}\qquad \end{aligned}$$

where again we have used matrix notation to indicate the structure. This gives us an intuition for understanding the transformation properties under a ${\mathfrak {h}}\oplus {\mathfrak {g}}$ rotation:

3.4 $$\begin{aligned} \begin{aligned} \delta A_{{\mathfrak {e}}_8} ~&\rightarrow ~ \delta A_{{\mathfrak {e}}_8} + [T_{{\mathfrak {e}}_8} , \,\delta A_{{\mathfrak {e}}_8}] \\&=~ \delta A_{{\mathfrak {e}}_8} + \begin{pmatrix} [ T_{{\mathfrak {h}}},\, \delta A_{\mathfrak {h}}] &{} \quad T_{{\mathfrak {h}}} \,\Phi - \Phi \, T_{{\mathfrak {g}}} \\ T_{{\mathfrak {g}}}\, \Psi - \Psi \, T_{{\mathfrak {h}}} &{} \quad [ T_{{\mathfrak {g}}}, \,\delta B_{\mathfrak {g}}]. \end{pmatrix} \end{aligned} \end{aligned}$$

We see that fields transform under ${\mathfrak {g}}\oplus {\mathfrak {h}}$ as:

1. $\delta A_{\mathfrak {h}}$ transforms as $({{\varvec{1}}},\mathrm{ad }_{{\mathfrak {h}}})$, and $\delta B_{\mathfrak {g}}$ transforms as $(\mathrm{ad }_{\mathfrak {g}}, {{\varvec{1}}})$;
2. $\Phi$ is in the $({{\overline{\varvec{R}}}},{{\varvec{r}}})$ and is a $r\times R$ matrix. Column vectors are in the fundamental; row vectors the antifundamental. For example, $\Phi ^{1},\ldots ,\Phi ^R$ are each column vectors transforming in the ${{\varvec{r}}}$ of ${\mathfrak {h}}$.
3. $\Psi$ is in the $({{\varvec{R}}},{{\overline{\varvec{r}}}})$ and is a $R\times r$ matrix with $\Psi ^1,\ldots ,\Psi ^R$ row vectors and so in the ${{\overline{\varvec{r}}}}$ of ${\mathfrak {h}}$.
4. To preserve the structure ${\mathfrak {g}}\oplus {\mathfrak {h}}$, $\delta A_{\mathfrak {h}}$ has legs only on the CYM, while $\delta B_{\mathfrak {g}}$ has legs only in .

The reality condition is $\delta A_{{\mathfrak {e}}_8}^\dag = - \,\delta A_{{\mathfrak {e}}_8}$ which implies

$$\begin{aligned} \delta A_{\mathfrak {h}}^\dag = - \,\delta A_{\mathfrak {h}}, \qquad \Phi ^\dag = - \,\Psi , \qquad \delta B_{\mathfrak {g}}^\dag = - \,\delta B_{\mathfrak {g}}. \end{aligned}$$

Decomposing this condition according to the holomorphic type of  we have:

$$\begin{aligned} \delta A_{\mathfrak {h}}= \delta \mathcal {A}- \delta \mathcal {A}^\dag , \qquad \Phi = \phi - \psi ^\dag , \quad \Psi =\psi - \phi ^\dag , \end{aligned}$$

where

$$\begin{aligned} \phi = \Phi _{0,1}, \quad \psi = \Psi _{0,1}, \end{aligned}$$

while the (1, 0)-components are fixed by reality: $\Phi _{1,0} = -\psi ^\dag$, $\Psi _{1,0} = -\,\phi ^\dag$.^4^

The ${\mathfrak {e}}_8$ field strength is

$$\begin{aligned} F_{{\mathfrak {e}}_8} = \text {d}A_{{\mathfrak {e}}_8} + A_{{\mathfrak {e}}_8}^2. \end{aligned}$$

Its background value decomposes according to its orientation of legs:

3.5 $$\begin{aligned} \begin{aligned} F_{{\mathfrak {e}}_8 ef} \,\text {d}X^e \,\text {d}y^f&=\text {d}B_{\mathfrak {g}}+ B_{\mathfrak {g}}^2,\\ F_{{\mathfrak {e}}_8 mn} \,\text {d}x^m \,\text {d}x^n&= \text {d}A_{\mathfrak {h}}+ A_{\mathfrak {h}}^2,\\ F_{{\mathfrak {e}}_8 me} \,\text {d}x^m \,\text {d}X^e&= \left( \partial _m B_{{\mathfrak {g}}\,e} - \partial _e A_{{\mathfrak {h}}\,m} + A_{{\mathfrak {h}}\,m} B_{{\mathfrak {g}}\,e} - B_{{\mathfrak {g}}\,e} A_{{\mathfrak {h}}\,m}\right) \,\text {d}x^m \,\text {d}X^e = 0.\\ \end{aligned} \end{aligned}$$

Under $$A_{{\mathfrak {e}}_8} \rightarrow A_{{\mathfrak {e}}_8} + \delta A_{{\mathfrak {e}}_8}$$,

3.6 $$\begin{aligned} \begin{aligned} \delta F_{{\mathfrak {e}}_8}&= \text {d}_{A_{{\mathfrak {e}}_8}} \delta A_{{\mathfrak {e}}_8} \\&=~ \begin{pmatrix} \text {d}_A( \delta A_{\mathfrak {h}}) &{} \quad \text {d}\Phi + A_{\mathfrak {h}}\Phi + \Phi B_{\mathfrak {g}}\\ \text {d}\Psi + \Psi A_{\mathfrak {h}}+ B_{\mathfrak {g}}\Psi &{} \quad \text {d}_{B_{\mathfrak {g}}} (\delta B_{\mathfrak {g}}) \end{pmatrix}. \\ \end{aligned} \end{aligned}$$

Consider $\delta F_{{\mathfrak {e}}_8}$ oriented along . At this point we drop the ${\mathfrak {h}}, {\mathfrak {g}}$ subscripts on *A*, *B*. Then, the equations of motion require $F_{{\mathfrak {e}}_8}$ be (1, 1) implying any (0, 2)-component must satisfy,The off-diagonal terms tell us the fields $\phi ,\psi$ are holomorphic sections of . We will occasionally introduce index to make manifest the fact that $\phi$ is in the $({{\overline{\varvec{R}}}}, {{\varvec{r}}})$ of ${\mathfrak {g}}\oplus {\mathfrak {h}}$ by writing $\phi ^{i\,{\overline{M}}}$ where $i,{\bar{\jmath }}=1, \ldots , {r}$ for representations ${{\varvec{r}}}$ of ${\mathfrak {h}}$; $M,{\overline{N}}=1,\ldots , R$ for representations ${{\varvec{R}}}$ of ${\mathfrak {g}}$. Then, for example,3.7We have introduced the $\overline{\partial }$-cohomology group , with forms valued in the ${\mathfrak {h}}$-subbundle of  whose fibres are the representation ${{\varvec{r}}}$ of ${\mathfrak {h}}$. The ${{\varvec{r}}}$ index $i,{\bar{\jmath }}$ is implicitly summed.

We could also study the equations of motion for $\delta A_{{\mathfrak {e}}_8}$ $\text {d}^\dag _A (\text {d}_A \delta A_{{\mathfrak {e}}_8}) = 0.$ Choosing the gauge $\text {d}_A^\dag \delta A_{{\mathfrak {e}}_8} = 0$ we see $\Box _A \delta A_{{\mathfrak {e}}_8} = 0.$ For example, on the $\phi$ matter field this gives

$$\begin{aligned} \left( \overline{\partial }_\mathcal {A}\overline{\partial }_\mathcal {A}^\dag + \overline{\partial }_\mathcal {A}^\dag \overline{\partial }_\mathcal {A}\right) \phi = 0. \end{aligned}$$

This has solution if $\phi$ is harmonic element of . This is slightly stronger than the cohomology relation (3.7).

Expand the fields $\phi$ and $\psi$ in a harmonic basis for  and , respectively:3.8where  and  are harmonic forms3.9while $C^\xi$ and  are valued in ${{\overline{\varvec{R}}}}$ and ${{\varvec{R}}}$, respectively.

For example, consider the standard embedding. Then,  and ;  with the . $C^\xi$ and  are in the ${{\overline{\varvec{R}}}} = {{\overline{\varvec{27}}}}$ and ${{\varvec{R}}} = {{\varvec{27}}}$.

We need to satisfy the reality condition $\Phi ^\dag = -\Psi$, which forces $\phi ^\dag =- \psi$ and so in terms of the  basis:3.10We denote conjugation through the barring of the indices. For example, $\phi _{\overline{\xi }}= (\phi _\xi )^\dag$ is a (1, 0)-form valued in ${{\overline{\varvec{r}}}}$ of ${\mathfrak {h}}$ and $C^{\overline{\xi }}= (C^\xi )^\dag$ is in the ${{\varvec{R}}}$ of ${\mathfrak {g}}$.

### The matter field metric from reducing Yang-Mills, $\mathcal {L}_F$

The spirit of KK reduction is to promote the coefficients to spacetime fields: $Y^\alpha (X)$, , and integrate over the six-dimensional manifold to get an effective four-dimensional theory. With the conventions of [5], the $d=10$ ${\mathfrak {e}}_8$ Yang–Mills field contribution to the $d=4$ effective field theory is:3.11We dimensionally reduce, doing a background field expansion. A small fluctuation of the field strength is given by (3.6), and so

3.12 $$\begin{aligned} \begin{aligned} \,\text {Tr}\,|\delta F_{{\mathfrak {e}}_8}|^2&= \,\text {Tr}\,\left( \text {d}_{A}( \delta A )\,\star \,\text {d}_{A}( \delta A) \right) + \,\text {Tr}\,\left( \text {d}_{A+B} \Phi \, \star \,\text {d}_{A+B} \Psi \right) \\&\quad +\,\,\text {Tr}\,\left( \text {d}_{A+B} \Psi \,\star \, \text {d}_{A+B} \Phi \right) + \,\text {Tr}\,\left( \text {d}_{B} \delta B \, \star \,\text {d}_{B} \delta B \right) , \end{aligned} \end{aligned}$$

The first term involves just the bundle moduli, contributing to the moduli metric considered in [5]; the middle two terms involve the matter fields and the last term gives rise to the kinetic term for the $d=4$ spacetime gauge field. The terms involving the matter fields are:

3.13 $$\begin{aligned} \begin{aligned} \text {d}_{A+B} \Phi&= (\partial _e \Phi + \Phi B_e) \,\text {d}X^e + (\partial _M \Phi _N + A_M \Phi _N)\, \text {d}x^M \text {d}x^N \\&= \widehat{\mathcal {D}}_e \Phi \,\text {d}X^e+ \text {d}_A \Phi ,\\ \text {d}_{A+B} \Psi&= \widehat{\mathcal {D}}_e \Psi \, \text {d}X^e + \text {d}_A \Psi ,\\ \end{aligned} \end{aligned}$$

where $\widehat{\mathcal {D}}_e$ is the spacetime ${\mathfrak {g}}$-covariant derivative and $\text {d}_A$ the ${\mathfrak {h}}$-covariant derivative. Hence, using $\,\text {Tr}\,|\delta F|^2 = \frac{1}{2}\,\text {Tr}\,\delta F_{MN} \delta F^{MN} = 2 \,\text {Tr}\,\delta F_{e\mu } \delta F^{e\mu },$ where $\,\text {Tr}\,(\delta F_{e\mu } \delta F^{e\mu } ) = \,\text {Tr}\,(\delta F_{e{\bar{\nu }}} \delta F^{e{\bar{\nu }}})$, and ignoring the moduli fields for the moment, we find the kinetic terms for the matter fields come from middle two terms in (3.12) and are

3.14 $$\begin{aligned} \begin{aligned} \,\text {Tr}\,|\delta F_{{\mathfrak {e}}_8}|^2 = - 2 \,\text {Tr}\,\left( \widehat{\mathcal {D}}_e \Phi _{\bar{\mu }}\, \widehat{\mathcal {D}}^e \Phi ^{\dagger \,{\bar{\mu }}}\right) - 2\,\text {Tr}\,\left( \widehat{\mathcal {D}}_e \Psi _{\bar{\mu }}\, \widehat{\mathcal {D}}^e \Psi ^{\dagger \,{\bar{\mu }}}\right) . \end{aligned} \end{aligned}$$

We have used the reality condition $\Phi ^\dag = -\Psi$. The matter fields have a KK ansatz, given by (3.10), which when substituted into each of the above terms gives3.15where indices for the representation ${{\varvec{R}}}$ and ${{\varvec{r}}}$ are explicit. The trace projects onto invariants constructed by the Krönecker delta functions $\delta _{i{\bar{\jmath }}}$ and $\delta _{M{\overline{N}}}$. In the following we will suppress the indices and delta symbols where confusion will not arise.

Substituting (3.14) and (3.15) into $\mathcal {L}_F$ in (3.11), reintroducing the moduli contribution, calculated in [5], we find a kinetic term for both the matter fields and the moduli fields:

3.16 $$\begin{aligned} \begin{aligned} \mathcal {L}_{F}&= -2G_{\alpha {\overline{\beta }}} \partial _e Y^\alpha \,\partial ^e Y^{\overline{\beta }}- 2G_{\xi {\overline{\eta }}} \, \widehat{\mathcal {D}}_e C^{\xi }\, \widehat{\mathcal {D}}^e C^{{\overline{\eta }}} - 2G_{\sigma {\overline{\tau }}} \, \widehat{\mathcal {D}}_e D^{{\overline{\tau }}}\, \widehat{\mathcal {D}}^e D^{\sigma }, \end{aligned} \end{aligned}$$

from which we may identify the moduli space metric and matter field metric

3.17 $$\begin{aligned} \text {d}s^2 = 2G_{\alpha {\overline{\beta }}} \text {d}y^\alpha \,\text {d}y^{\overline{\beta }}+ 2G_{\xi {\overline{\eta }}} \, \text {d}C^{\xi }\text {d}C^{{\overline{\eta }}} + 2G_{\sigma {\overline{\tau }}} \, \text {d}D^{\sigma }\, \text {d}D^{{\overline{\tau }}}, \end{aligned}$$

where we denote the coordinates of the moduli space $\mathcal {M}$ by $y^\alpha , y^{\overline{\beta }}$, and without wanting to clutter formulae, denote the coordinates of the matter fields by $C^\xi , D^\sigma$—any ambiguity with their corresponding fields will always be made explicit. The moduli fields have the metric computed in [5], given in (5.2). The matter fields also have a metric3.18There is no trace as the integrands are written in the form ${{\overline{\varvec{r}}}}\cdot {{\varvec{r}}}$. Although we have indicated the result for a single representation ${{\varvec{r}}}$ and ${{\overline{\varvec{r}}}}$, the result generalises to a sum over representations of ${\mathfrak {h}}$.

For any two-form $\mathcal {F}$ there is a relation $\omega \star \mathcal {F}= \frac{1}{2}\mathcal {F}\omega ^2.$ Using this and $\omega ^{\mu {\bar{\nu }}} = -\text {i}g^{\mu {\bar{\nu }}}$ we find

3.19 $$\begin{aligned} \begin{aligned} \,\phi _{\overline{\eta }}\star \phi _\xi&=-\text {i}\omega \star \phi _\xi ^i \, \phi ^{\bar{\jmath }}_{\overline{\eta }}\, \delta _{i{\bar{\jmath }}} =- \frac{\text {i}}{2} \omega ^2 \,\,\text {Tr}\,(\phi _\xi \,\phi _{\overline{\eta }}),\\ \,\psi _\sigma \star \psi _{\overline{\tau }}&=-\text {i}\omega \star \psi _\sigma ^{\bar{\jmath }}\, \psi ^i_{\overline{\tau }}\, \delta _{i{\bar{\jmath }}}= -\frac{\text {i}}{2} \omega ^2 \,\,\text {Tr}\,(\psi _\sigma \,\psi _{\overline{\tau }}). \end{aligned} \end{aligned}$$

We introduce the trace over ${{\varvec{r}}}$ indices in order to be able to write $\phi _x, \psi _\sigma$ in any order. The matter field metrics are then expressible in a way closely resembling the moduli metric3.20


## Fermions and Yukawa couplings

The fermionic couplings of interest to heterotic geometry derive from the kinetic term for the gaugino. We compute the quadratic and cubic fluctuation terms. The former are mass terms for the gauginos, which we show all vanish consistent with the vacuum being supersymmetric. The latter are the Yukawa couplings between two gauginos and a gauge boson.

In “Appendix B” all spinor conventions we used are explained. We also give a summary of results in spinors in $d=4,6,10$ relevant to this section. We also derive some expressions for bilinears relevant to the dimensional reduction.

### Fermion zero modes on 

#### ${\mathfrak {so}}(3,1)\oplus {\mathfrak {su}}(3)$ spinors

The gaugino is a Majorana–Weyl spinor $\varepsilon$ which has zero modes on . The Lorentz algebra is ${\mathfrak {so}}(3,1)\oplus {\mathfrak {so}}(6) \subset {\mathfrak {so}}(9,1)$ under which $\varepsilon$ is

$$\begin{aligned} \varepsilon = \zeta ' \otimes \lambda '\oplus \zeta \otimes \lambda ~\cong ~ \begin{pmatrix} \zeta '\\ 0 \end{pmatrix}\otimes \lambda ' + \begin{pmatrix} 0\\ {\overline{\zeta }}\end{pmatrix}\otimes \lambda , \end{aligned}$$

where $\lambda$ and $\lambda '$ are in the ${{\varvec{4}}}$ and ${{\varvec{4}}}'$ of ${\mathfrak {so}}(6)$; $\zeta$ and $\zeta '$ are in the ${{\varvec{2}}}$ and ${{\varvec{2}}}'$ of ${\mathfrak {so}}(3,1)$. Where possible we use the 2-component Weyl notation for $\zeta ,\zeta '$ and always leave the ${\mathfrak {so}}(6)$ spinor indices implicit. We write $\oplus$ in this context to reflect the embedding of 2-component spinors into a 4-component notation as shown by the second equality. The barring of 2-component spinors, and dotting the spinor index, comes with complex conjugation $(\zeta ^a)^* = {\overline{\zeta }}^{\dot{a}}$ as described in appendix. The fermions $\zeta ,\zeta '$ are Grassmann odd and under complex conjugation are interchanged without paying the price of a sign.

The Majorana condition implies $\zeta \otimes \lambda$ are determined in terms of $\zeta '\otimes \lambda '$. With our conventions this means

4.1 $$\begin{aligned} {\overline{\zeta }}^{\dot{a}}\otimes \lambda = {\overline{\zeta }}'{}^{\dot{a}}\otimes \lambda '^c, \end{aligned}$$

where $\lambda '^c$ denotes taking the ${\mathfrak {so}}(6)$ Majorana conjugate. The Majorana–Weyl spinor $\varepsilon$ can now be written solely in terms of say $\zeta ',\lambda '$:

4.2 $$\begin{aligned} \varepsilon = \begin{pmatrix} \zeta _a'\\ 0 \end{pmatrix}\otimes \lambda '~ + \begin{pmatrix} 0\\ {\overline{\zeta }}'^{\dot{a}}\end{pmatrix}\otimes \lambda '{}^c . \end{aligned}$$

The presence of $\mathcal {N}=1$ spacetime supersymmetry means there is a globally well-defined spinor on . This implies the existence of an ${\mathfrak {su}}(3)$-structure on . Under ${\mathfrak {so}}(6) \rightarrow {\mathfrak {su}}(3)$ the spinors $\lambda ,\lambda '$ decompose according to the branching rule ${{\varvec{4}}} = {{\varvec{3}}} \oplus {{\varvec{1}}}$, and ${{\varvec{4}}}' = {{\overline{\varvec{3}}}} \oplus {{\varvec{1}}}$, which we write as

$$\begin{aligned} \lambda = \lambda _{{{\varvec{3}}}} \oplus \lambda _+,\qquad \qquad \lambda ' = \lambda _{{{\overline{\varvec{3}}}}} \oplus \lambda _-. \end{aligned}$$

The spinors $\lambda _+, \lambda _-$ are the nowhere vanishing ${\mathfrak {su}}(3)$ invariant spinors. As established in appendix, we can express $\lambda _{{{\varvec{3}}}}, \lambda _{{{\overline{\varvec{3}}}}}$ in terms of $\lambda _\pm$ and gamma matrices

4.3 $$\begin{aligned} \lambda _{{{\varvec{3}}}} = \Lambda _\mu \gamma ^\mu \lambda _-, \qquad \lambda _{{{\overline{\varvec{3}}}}} = \Lambda '_{\bar{\mu }}\gamma ^{\bar{\mu }}\lambda _+, \end{aligned}$$

where $\{\gamma ^\mu , \gamma ^{\bar{\nu }}\} = g^{\mu {\bar{\nu }}}$ and $\Lambda _\mu , \Lambda '_{\bar{\mu }}$ are components of 1-forms on . Appendix details the construction of the ${\mathfrak {su}}(3)$ bilinears:

4.4 $$\begin{aligned} \lambda _+^\dag \gamma ^\mu \gamma ^{\bar{\nu }}\lambda _+ = g^{\mu {\bar{\nu }}}, \qquad \lambda _-^\dag \gamma ^{\bar{\nu }}\gamma ^\mu \lambda _- = g^{\mu {\bar{\nu }}}, \end{aligned}$$

and

4.5 $$\begin{aligned} \Omega _{\mu \nu \rho }= - e^{-\text {i}\phi }\,||\Omega ||\, \lambda _-^\dag \gamma _{\mu \nu \rho } \lambda _+,\qquad {\overline{ \Omega }}_{\overline{\mu \nu \rho }}= e^{\text {i}\phi }\,||\Omega ||\, \lambda _+^\dag \gamma _{\overline{\mu \nu \rho }} \lambda _-, \end{aligned}$$

where $\phi$ accounts for a relative phase difference between $\lambda _-$ and $\Omega$. Under the gauge symmetry $\Omega \rightarrow \mu \Omega$, the fermions $\lambda _\pm$ transform as a phase:

$$\begin{aligned} \lambda _\pm \rightarrow e^{\pm \text {i}\xi /2} \lambda _\pm , \quad \mu = |\mu | e^{\text {i}\xi }. \end{aligned}$$

The bilinears above respect this gauge symmetry.

Given (4.3), the action of Majorana conjugation is

4.6 $$\begin{aligned} \begin{aligned} \lambda _\pm ^c&= -\text {i}\lambda _\mp , \qquad \lambda _{{{\overline{\varvec{3}}}}}^c = \text {i}\Lambda '{}_\mu ^\dag \gamma ^\mu \lambda _-, \qquad \lambda _{{{\varvec{3}}}}^c = \text {i}\Lambda _{\bar{\mu }}^\dag \gamma ^{\bar{\mu }}\lambda _+. \end{aligned} \end{aligned}$$

#### Kaluza Klein ansatz

$\varepsilon$ is in the adjoint of ${\mathfrak {e}}_8$. Consequently, it decomposes under ${\mathfrak {g}}\oplus {\mathfrak {h}}\subset {\mathfrak {e}}_8$ and the expectation from supersymmetry is that we find a natural pairing between fluctuations of the gauge field and the fermions. As the background is bosonic, all fermionic fields are fluctuations; we aim to study the effective field theory of those fluctuations that are massless. The massless fluctuations are zero modes of an appropriate Dirac operator.

The gaugino and gauge field have decomposition under ${\mathfrak {so}}(3,1) \oplus {\mathfrak {su}}(3) \subset {\mathfrak {so}}(9,1)$

4.7 $$\begin{aligned} \begin{aligned} \varepsilon :\quad {{\varvec{16}}}&= \left( {{\varvec{2}}} \otimes {{\varvec{1}}} \oplus {{\varvec{2}}}'\otimes {{\varvec{1}}}\right) \oplus \left( {{\varvec{2}}}\otimes {{\varvec{3}}} \right) \oplus \left( {{\varvec{2}}}'\otimes {{\overline{\varvec{3}}}}\right) ,\\A:\quad {{\varvec{10}}}&= {{\varvec{4}}} \oplus {{\varvec{3}}} \oplus {{\overline{\varvec{3}}}}, \end{aligned} \end{aligned}$$

and for the gauge algebra ${\mathfrak {e}}_8 \supset {\mathfrak {g}}\oplus {\mathfrak {h}}$:

$$\begin{aligned} {\mathrm{ad}}_{{\mathfrak {e}}_8} = ({{\varvec{1}}}, {\mathrm{ad}}_{{\mathfrak {h}}}) \oplus ({\mathrm{ad}}_{{\mathfrak {g}}}, {{\varvec{1}}}) \oplus _i ({{\overline{\varvec{R_i}}}}, {{\varvec{r_i}}}) \oplus _i({{\varvec{R_i}}}, {{\overline{\varvec{r_i}}}}). \end{aligned}$$

We organise our study of the zero modes according to their representations under ${\mathfrak {so}}(3,1)\oplus {\mathfrak {su}}(3)$ and the gauge algebra ${\mathfrak {g}}\oplus {\mathfrak {h}}$. We continue the mnemonic of indicating the gauge structure through block matrices

4.8 $$\begin{aligned} \begin{aligned} \delta A&= \delta A_{(\mathrm{ad }_{\mathfrak {g}},{{\varvec{1}}})} \oplus _i \delta A_{({{\varvec{R_i}}},{{\overline{\varvec{r_i}}}})}\oplus _j \delta A_{({{\overline{\varvec{R_j}}}},{{\varvec{r_j}}})} \oplus \delta A_{({{\varvec{1}}}, \mathrm{ad }_{\mathfrak {h}})} \\&=~ \begin{pmatrix} \delta A_{\mathrm{ad }_{\mathfrak {h}}} &{} \quad \oplus _j \delta A_{({{\overline{\varvec{R_j}}}},{{\varvec{r_j}}})} \\ \oplus _i\delta A_{({{\varvec{R_i}}},{{\overline{\varvec{r_i}}}})} &{} \quad \delta A_{\mathrm{ad }_{{\mathfrak {g}}}} \end{pmatrix} = \begin{pmatrix} \delta \mathcal {A}- \delta \mathcal {A}^\dag &{} \quad \Phi \\ \Psi &{} \quad \delta B \end{pmatrix}, \\ \varepsilon&=~ \varepsilon _{(\mathrm{ad }_{\mathfrak {g}},{{\varvec{1}}})} \oplus _i \varepsilon _{({{\varvec{R_i}}},{{\overline{\varvec{r_j}}}})}\oplus _j \varepsilon _{({{\overline{\varvec{R_j}}}},{{\varvec{r_j}}})} \oplus \varepsilon _{({{\varvec{1}}}, \mathrm{ad }_{\mathfrak {h}})}\\&=~ \begin{pmatrix} ( \zeta '\otimes \lambda ' \oplus \zeta '{}^c\otimes \lambda '{}^c)_{({{\varvec{1}}},\mathrm{ad }_{\mathfrak {h}})} &{}\quad \oplus _j( \zeta '\otimes \lambda ' \oplus \zeta '{}^c\otimes \lambda '{}^c)_{({{\overline{\varvec{R_j}}}},{{\varvec{r_j}}})} \\ \oplus _i ( \zeta '\otimes \lambda ' \oplus \zeta '{}^c\otimes \lambda '{}^c)_{({{\varvec{R_i}}},{{\overline{\varvec{r_i}}}})} &{} \quad (\zeta '\otimes \lambda ' \oplus \zeta '{}^c\otimes \lambda '{}^c)_{(\mathrm{ad }_{\mathfrak {g}},{{\varvec{1}}})} \end{pmatrix}. \end{aligned} \end{aligned}$$

In the second line we have indicated the representations of the individual components of the gaugino by a subscript, and these are regarded as independent field fluctuations. We will now drop the sum over representations $\oplus _i$ in order to simplify notation and as the generalisation to include a sum over representations is obvious.

We classify the zero modes by the symmetries under ${\mathfrak {su}}(3)$-structure and ${\mathfrak {g}}\oplus {\mathfrak {h}}$. The first type of zero modes is ${\mathfrak {su}}(3)$-structure singlets and transforms in the $(\mathrm{ad }_{\mathfrak {g}},{{\varvec{1}}})$ with KK ansatz

4.9 $$\begin{aligned} \begin{aligned} \delta A_{{\mathfrak {e}}_8\,e}\, \text {d}X^e&= \begin{pmatrix} 0 &{} \quad 0 \\ 0 &{}\quad \delta B_e \text {d}X^e \end{pmatrix},\\ \varepsilon _{ (\mathrm{ad }_{\mathfrak {g}},{{\varvec{1}}})}&= \begin{pmatrix} 0 &{} \quad 0\\ 0 &{} \quad \zeta _{\mathrm{ad }_{\mathfrak {g}}} \end{pmatrix} \otimes \lambda _- \oplus \begin{pmatrix} 0 &{} \quad 0\\ 0 &{} \quad \text {i}{\overline{\zeta }}_{\mathrm{ad }_{\mathfrak {g}}} \end{pmatrix} \otimes \lambda _+, \end{aligned}~ \end{aligned}$$

where we use the first line of (B.36).

The second type of zero modes transforms as ${{\overline{\varvec{3}}}}$ under ${\mathfrak {su}}(3)$-structure and as $({{\varvec{1}}},\mathrm{ad }_{{\mathfrak {h}}}) \oplus ({{\varvec{R}}},{{\overline{\varvec{r}}}}) \oplus ({{\overline{\varvec{R}}}},{{\varvec{r}}})$ under ${\mathfrak {g}}\oplus {\mathfrak {h}}$. There is a natural pairing between $\delta A^{0,1}_{{\mathfrak {e}}_8}$ and $\zeta '\otimes \lambda _{{{\overline{\varvec{3}}}}}$. Using the KK ansatz (3.3), (3.10) for bosons there is a corresponding KK ansatz for the spinors:4.10where the matrices in the last line are related to the $\Lambda _{\bar{\mu }}'$ in (4.3). We have denoted the anticommuting  spinors by calligraphic letters $\mathcal {Y}^\alpha$, $\mathcal {C}^{\xi }$ and ; superpartners to . $\mathcal {C}^{\xi }$ and  are in the ${{\overline{\varvec{R}}}}$ and ${{\varvec{R}}}$ of ${\mathfrak {g}}$, while $\mathcal {Y}^\alpha$ are neutral.

The spinor in the ${{\varvec{3}}}$ of ${\mathfrak {su}}(3)$-structure is determined by Majorana conjugation ${\overline{\zeta }}^{\dot{a}}\otimes \lambda _{{{\varvec{3}}}} = {\overline{\zeta }}'^\dag {}^{\dot{a}}\otimes \lambda _{{{\overline{\varvec{3}}}}}^c$, expressed through (4.1) and (4.6):4.11Altogether, the Majorana–Weyl spinor is given by substituting the above two expressions into the first line of (B.37) and combining with (4.9) giving4.12The $\oplus$ reflects the embedding of the 2-component Weyl spinors into a 4-component notation used in (B.36), (B.37).

### Dimensional reduction of 

The ten-dimensional kinetic term for the gaugino is now dimensionally reduced, with action: 4.13The quadratic fluctuations give the kinetic terms as well as any mass terms; the cubic fluctuations give Yukawa interactions.

We split the bilinear into two terms4.14where  is the Dirac conjugate.  are the $d=10$ gamma matrices, given asas described in appendix. Here $\mu$ is a holomorphic index along .

#### Quadratic couplings

As the background is bosonic, we take *A* to be the background gauge field

4.15 $$\begin{aligned} A = \begin{pmatrix} \mathcal {A}- \mathcal {A}^\dag &{} \quad 0 \\ 0 &{} \quad B_e \text {d}X^e \end{pmatrix}. \end{aligned}$$

We start with the derivative operatorThe first term  is computed using (4.2) and the third lines of (B.36), (B.37),4.16Spinor and representation indices are contracted in the natural way.

The second term  follows from the fourth lines of (B.36), (B.37) together with the relation in (A.5):4.17Next we compute the reduction of4.18The first terms follows the calculation of (4.17) after using (4.15) and $\,\text {Tr}\,B_e = 0$
4.19The second term  mirrors the calculation of (4.20), using the background (4.15)4.20We now put the terms together. The  term comes from adding (4.17) and (4.19):4.21where ${\hat{D}}_e$ is a spacetime covariant derivative appropriate to whatever representation if it acts onThe matter and moduli fields have kinetic terms with non-trivial metrics:4.22The fermions have identical metrics to their bosonic superpartners. The bundle moduli appear with a metric that coincides with that derived by Kobayashi and Itoh [19, 20].

The mass terms come from adding together (4.17) and (4.20). We normalise the mass term to be compatible with the convention in [31]:4.23where  is the Kähler potential, which on our background evaluates to be . The mass terms are4.24The last term is normalised with a factor of 2 as the two indices are distinguished. As before, we do not write the trace, understanding the indices contracted in the natural way.

Recall that the equations of motion are4.25Substituting this in we find4.26The vanishing of $m_{\alpha \beta }$ is guaranteed when , that is for bundle or Hermitian moduli. For complex structure parameters, if one can find a basis for parameters in which  for complex structure moduli, so that $\Delta _\alpha {}^\mu F_\mu = 0$, then this is also satisfied. If this is not possible, we exploit the last line of the supersymmetry relation (2.29)4.27We have used some results familiar from special geometry, see [21], which apply in this general heterotic context. They are:Here $\mathcal {D}_\alpha \chi _\beta$ is covariant with respect to gauge transformations $\chi _\alpha \rightarrow \mu \, \chi _\alpha$. Also,

$$\begin{aligned} \mathfrak {a}_{\alpha \beta \gamma } = - \int \Omega \Delta _\alpha {}^\mu \Delta _\beta {}^\nu \Delta _\gamma {}^\rho \ \Omega _{\mu \nu \rho }, \qquad \mathfrak {a}_{\alpha \beta }{}^{\overline{\gamma }}= \mathfrak {a}_{\alpha \beta \gamma } G_{0}{}^{\gamma {\overline{\gamma }}}, \quad G_{0\,\alpha {\overline{\beta }}} ~=-\frac{\int \chi _\alpha \chi _{\overline{\beta }}}{\int \Omega {\overline{ \Omega }}}. \end{aligned}$$

andIn special geometry $\mathfrak {a}_{\alpha \beta \gamma }$ is a Yukawa coupling for ${{\varvec{27}}}^3$ fields, playing the role of an intersection quantity relating derivatives of $\chi _\alpha$ to $\overline{\chi }_{\overline{\gamma }}$. $G_0$ the metric on complex structures, used to raise and lower indices. The same relation applies in heterotic geometry with the understanding that the complex structures may be reduced by the Atiyah constraint. Consequently, we see that the Atiyah condition gives $m_{\alpha \beta } = 0.$

#### Cubic fluctuations and Yukawa couplings

We now compute the cubic order fluctuations to get the Yukawa couplings. The calculation proceeds in a similar fashion to the above. The cubic interaction only comes from:4.28The fluctuations only occur on the internal space . The gauge structure of $\delta A$ is specified in (4.8). The calculation is a simple generalisation of the result (4.20) using the fourth line of (B.37).4.29In the last two lines, we use the appropriate symmetric invariants to construct ${{\varvec{R}}}^3$ and ${{\overline{\varvec{R}}}}^3$.

Putting it together, normalising to agree with [31], we find4.30where $\mathrm{c.c}$ denotes the complex conjugate and the Yukawa couplings are given by4.31The result is straightforwardly extended to vacua with more complicated branching rules involving multiple representations $\oplus _p {{\varvec{R}}}_p$. More Yukawa couplings appear—one for each invariant computed via the trace—but the integrand is of the same form as above.

The ${{\varvec{1}}}^3$ coupling vanishes classically. To see this, write $\delta \mathcal {A}$ to second order in deformationsNote that  and so the second term is appropriately symmetric in indices $\alpha ,\beta$. A standard deformation theory argument related to the Kuranishi map implies the second-order deformation is unobstructed providedSubstituting into (5.4) one finds  when $\Delta _\alpha {}^\mu F_\mu = 0$. When $\Delta _\alpha \ne 0$, that is complex structure is varying, the coupling still vanishes with exactly the same argument as for the singlet mass term in (4.27).

The coupling  also vanishes by demanding that $\phi _\xi$, equivalently, , remain solutions of the equation of motion under a bundle deformation : . Hence, the singlet couplings vanish.


## The final result: moduli, matter metrics and Yukawa couplings

The effective field theory has $\mathcal {N}=1$ supersymmetry, with a gravity multiplet and a gauge symmetry ${\mathfrak {g}}$. The $\mathcal {N}=1$ chiral multiplets consist of 5

- ${\mathfrak {g}}$-neutral scalar fields $Y^\alpha$ and fermions $\mathcal {Y}^\alpha$ corresponding to moduli;
- ${\mathfrak {g}}$-charged bosons $C^\xi$ and fermions $\mathcal {C}^\xi$ in the ${{\overline{\varvec{R}}}}$ of ${\mathfrak {g}}$;
- ${\mathfrak {g}}$-charged bosons $D^\rho$ and fermions $\mathcal {D}^\rho$ in the ${{\varvec{R}}}$ of ${\mathfrak {g}}$;

The final result is expressed as a Lagrangian with normalisation conventions matching [31]5.1The kinetic terms for fields contain metrics. The metric for fermions and bosons are identical, consistent with supersymmetry. The moduli metric, derived in [5], is:

5.2 $$\begin{aligned} \begin{aligned} \text {d}s^2&= 2G_{\alpha {\overline{\beta }}} \,\text {d}y^\alpha \otimes \text {d}y^{\overline{\beta }}, \\ G_{\alpha {\overline{\beta }}}&= \frac{1}{4V} \int \Delta _\alpha {}^\mu \star \Delta _{\overline{\beta }}{}^\nu \,\, g_{\mu {\bar{\nu }}} + \frac{1}{4V} \int \mathcal {Z}_\alpha \star \mathcal {Z}_{\overline{\beta }}\,+ \\&\quad + \,\frac{ {\alpha ^{\backprime }\,}}{4V}\int \,\text {Tr}\,\Big ( D_\alpha A \star D_{\overline{\beta }}A \Big ) - \frac{ {\alpha ^{\backprime }\,}}{4V}\int \,\text {Tr}\,\Big (D_\alpha \Theta \,\star \, D_{\overline{\beta }}\Theta ^\dag \Big ). \end{aligned} \end{aligned}$$

The metric terms for the fermionic superpartners to moduli $\mathcal {Y}^\alpha$ are fixed by supersymmetry from the bosonic result. The matter field metrics are given in (3.20),5.3The mass terms written in (4.24) vanish 

The Yukawa nonzero couplings in (4.31) are5.4


## The superpotential and Kähler potential

The effective field theory has $\mathcal {N}=1$ supersymmetry in , and so the couplings ought to be derivable from a superpotential and Kähler potential. The Kähler potential for the moduli metric couplings was proposed in [5], and checked against a dimensional reduction of the ${\alpha ^{\backprime }\,}$-corrected supergravity action. It is6.1in which $\omega$ is the Hermitian form of . The ${\alpha ^{\backprime }\,}$-corrections preserved the form of the special geometry Kähler potential, and the second term remains classical.

The Kähler potential for the matter field metric is trivial and given by6.2where $a,b=1,\ldots , R$ label the ${{\varvec{R}}}$ representation and the trace is taken with respect to the delta function.

The F-term couplings for the $d=4$ chiral multiplets are described by a superpotential. In the language of $d=4$ effective field theory, this superpotential takes the general form6.3where the $\,\text {Tr}\,$ projects onto the appropriate ${{\varvec{R}}}$-invariant and we are to view these as chiral multiplets in $N=1$ $d=4$ superspace in the usual way. The omitted terms are the quartic and higher-order couplings and non-perturbative corrections. It is important that  gives no singlet couplings, and this means all parameter derivatives of  vanish.

We would like to study a superpotential in a similar vein to the Kähler potential proposal (6.1). As ten-dimensional fields $A_{{\mathfrak {e}}_8}$ and *H* depend on both parameters and matter fields. The fields $\text {d}^{\text{ c }}\omega$ and $\Omega$ are valued on  and depend only on moduli fields. The spirit of the dimensional reduction is to promote the parameters to $d=4$ fields. In this vein define a superpotential 6
6.4in which the fields are regarded as functionals of the $d=4$ chiral multiplets. The couplings in the effective field theory are specified by differentiating  and evaluating the integral after fixing the parameters $y=y_0$.

The rules for differentiating fields in the expressions for  and  with respect to parameters have been described in [5], which is complicated by virtue of ${\mathfrak {h}}$ gauge transformations being parameter- and coordinate-dependent. These transformations are, however, independent of matter fields, and so the rule for matter field differentiation is simple

$$\begin{aligned} \partial _\xi A_{{\mathfrak {e}}_8} = \frac{\partial A_{{\mathfrak {e}}_8}}{\partial C^\xi } = \phi _\xi . \end{aligned}$$

It is important that we have written the ten-dimensional ${\mathfrak {e}}_8$ gauge field $A_{{\mathfrak {e}}_8}$, and not $A_{\mathfrak {h}}$, as this is the functional of the matter fields——as illustrated in, for example, (3.3) and (3.10). The integrand in  is a functional of the ten-dimensional *H* so that it depends on matter fields. The rule is to differentiate as noted above and then evaluate the integral on the fields’ vacuum expectation values (VEV). Note that it is the VEV of *H* that satisfies $\text {d}^{\text{ c }}\omega = H$, and the matter fields VEVs vanish .

For example, the tadpole matter and moduli couplings for a vacuum at the point $y=y_0$ are6.5where we use $\partial _\alpha H$ in (2.20) and $\partial _\alpha \text {d}^{\text{ c }}\omega$ in (2.26), and we evaluate them on some fixed $y=y_0$.

As an ansatz  must satisfy a number of tests: it must be a section of a line bundle over the moduli space; any derivative with respect to parameters must vanish viz. ; be a holomorphic function of chiral fields; tadpole and mass terms for the matter fields must vanish; capture the F-term couplings derived through dimensional reduction in this paper. The expression (6.4) passes these tests.^7^

 is a section of the line bundle transforming under the gauge symmetry $\Omega \rightarrow \mu (y) \Omega$ as  where $\mu (y)$ is a holomorphic function of parameters. This is necessary in order to consistently couple to gravity [22]. This fixes the integrand to be proportional to $\Omega$.

The supersymmetry relation $H= \text {d}^{\text{ c }}\omega$ holds for all $y_0\in \mathcal {M}$. Hence, derivatives of it vanish:^8^

6.6 $$\begin{aligned} \partial _{\alpha _1} \cdots \partial _{\alpha _n} (H-\text {d}^{\text{ c }}\omega )|_{y=y_0}= 0, \qquad \partial _{{\overline{\beta }}_1} \cdots \partial _{{\overline{\beta }}_n} (H-\text {d}^{\text{ c }}\omega )|_{y=y_0} = 0, \end{aligned}$$

where $y^{\alpha _1}, \ldots , y^{\alpha _n}$ are any collection of parameters and we evaluate on a supersymmetric vacuum, denoted by $y=y_0$. It then follows that any derivative of the superpotential with respect to parameters vanishes. This is what is used in (6.5) to show that all tadpole terms vanish. The argument clearly extends to higher order. Consider the *k*th derivativeThis vanishes on any supersymmetric background:  is independent of moduli fields, and so  does not give rise to any singlet couplings in agreement with the dimensional reduction.

An analogous argument, together with $\Omega$ being holomorphic, shows that despite neither *H* nor $\text {d}^c\omega$ being holomorphic,  is a holomorphic function of fields. For example, the first-order derivative isUsing (6.6) all higher-order antiholomorphic derivatives of $\Omega (H-\text {d}^{\text{ c }}\omega )$ vanish. It is also the case that  for all $n\ge 1$. So,  is a holomorphic function of chiral fields.

The expression for the masses can be written as derivatives of *W*
6.7where for the second term we use that $\text {d}^c \omega , \Omega$ do not depend on , while  is given by (2.22) with $D_a A \rightarrow \partial _\xi A = \phi _\xi$. As *A* depends linearly on the matter fields, all second derivatives vanish.

The Yukawa couplings  are also all derived from . Using (2.25), we find agreement with the functional forms in (4.31), of which the non-vanishing terms are6.8Even though the singlet couplings vanish, one can check that their functional form is correctly derivable from . The fact of 1 / 2 is in order to agree with the convention given in [31]. It is satisfying that the superpotential consistently captures the couplings derived in the dimensional reduction, both involving moduli and matter fields. Furthermore, it manifestly does not give rise to any singlet couplings.

## Outlook

We have calculated the effective field theory of heterotic vacua of the form  at large radius, correct to order ${\alpha ^{\backprime }\,}$. The field theory is specified by a Kähler potential and superpotential. Supersymmetry forbids  from being corrected perturbatively in ${\alpha ^{\backprime }\,}$, but is in general corrected non-perturbatively in ${\alpha ^{\backprime }\,}$. For  obtained by deforming , some of these non-perturbative corrections have been computed as functions of moduli using linear sigma models, see, for example, [23–27]. One can now use the results obtained here and those in [5] to determine the normalised quantum corrected Yukawa couplings, in examples that may be of phenomenological interest, see, for example, [28]. Although the Kähler potential is corrected perturbatively in ${\alpha ^{\backprime }\,}$, it was conjectured in [5] that the form of the Kähler potential does not change to all orders in perturbation theory, and that the ${\alpha ^{\backprime }\,}$-corrections are contained within the Hermitian form $\omega$. This conjecture is consistent with the work in [6, 7], and it would be very interesting to prove this conjecture, at least to second order in ${\alpha ^{\backprime }\,}$.

Although we have derived this result using a single pair of matter fields, the result clearly generalises to a sum over representations $\oplus _p {{\varvec{R}}}_p \oplus _p {{\overline{\varvec{R}}}}_p$. The main burden of the generalisation is to evaluate the trace using the appropriate branching rules.

Many questions arise. For example, are there any special geometry type relations between  and ? Finding a prepotential analogous to special geometry looks difficult, partly because it involved analysis related on the geometry of the standard embedding and Calabi–Yau manifold ’s. Nonetheless, it is likely  and  are related.

It would be interesting to compute the field theory couplings in specific examples. For  attained by deforming  one might be able to compare with the linear sigma model parameter space studied in say [24, 29, 30] and study the quantum corrections to the ${{\varvec{27}}}^3$ and ${{\overline{\varvec{27}}}}^3$ couplings using the correctly normalised fields. We showed using deformation theory arguments that the ${{\varvec{1}}}^3$ coupling vanishes classically. A pressing question is to what extent these couplings vanish exactly. Any non-vanishing would imply the vacuum does not exist, and thereby shrink the moduli space of heterotic vacua quantum mechanically.

## A Hodge theory on real and complex manifolds

We establish some notation and results for forms on real and complex manifolds to be used in the text. Coordinates for  are denoted $X^e$, while real coordinates on  are denoted by $x^m$. Complex coordinates are denoted $x^\mu , x^{\bar{\nu }}$.

We need to write coordinate expressions for forms more than metrics, and so our convention is to omit the wedge symbol $\wedge$ except where confusion may arise. We write metrics as $\text {d}s^2 = g_{mn} \text {d}x^m \otimes \text {d}x^n$, only occasionally omitting the $\otimes$ only where confusion will not arise.

### A.1 Real manifolds

The volume form on a *n*-dimensional Riemannian manifold is

A.1 $$\begin{aligned} \text {d}^6x g^\frac{1}{2}= \star 1 = \frac{\sqrt{g}}{n!} \,\epsilon _{m_1\ldots m_{n}}\, \text {d}x^{m_1} \ldots \text {d}x^{m_{n}},\qquad g = |\det g_{mn}|. \end{aligned}$$

where $\epsilon _{12\cdots n} = \epsilon ^{12\cdots n} =1$ is the permutation symbol. The determinant of the metric is

$$\begin{aligned} g = \frac{1}{n!} \epsilon ^{p_1\cdots p_n} \epsilon ^{q_1\cdots q_n} g_{p_1q_1}\cdots g_{p_n q_n}. \end{aligned}$$

If $\omega$ is a top-form, then

$$\begin{aligned} \text {d}^6x g^\frac{1}{2}\, \,\frac{\sqrt{g}}{n!} \,\,\epsilon ^{m_1\ldots m_{n}} \omega _{m_1\ldots m_n} = \frac{1}{n!} \omega _{m_1\ldots m_n} \text {d}x^1 \ldots \text {d}x^n. \end{aligned}$$

The Hodge dual of a *p*-form $A_p$ is

$$\begin{aligned} \star A_p = \frac{\sqrt{g}}{p!(n-p)!} \epsilon ^{m_1\ldots m_p}{}_{n_1\ldots n_{n-p}} A_{m_1\ldots m_p} dx^{n_1} \ldots dx^{n_{n-p}}. \end{aligned}$$

The inner product of two *p*-forms is then

$$\begin{aligned} A_p {\,\wedge \,}\star B_p = \text {d}^6x g^\frac{1}{2}\,\left( \frac{1}{p!}\,\, A_{m_1 \ldots m_p}\,B^{m_1\ldots m_p} \right) . \end{aligned}$$

### A.2 Complex manifolds

On a complex manifold the metric is Hermitian

$$\begin{aligned} \text {d}s^2 = 2 g_{\mu {\bar{\nu }}} \,\text {d}x^\mu \otimes \text {d}x^{\bar{\nu }}\end{aligned}$$

with $\det (g_{mn}) {=} g$. In addition to the Hodge dual $\star$, which contracts a (*p*, *q*) with a (*q*, *p*) form, on a complex manifold we can define a $\,{\overline{ \star }\,}$ which contracts a pair of (*p*, *q*)-forms, and so forming an inner product. If $\alpha ,\beta$ are two (*p*, *q*)-forms, then it is defined as

A.2 $$\begin{aligned} \alpha \,{\overline{ \star }\,}\beta ~:=~\frac{1}{p!q!}\, \alpha _{\mu _1\cdots \mu _p {\bar{\nu }}_1\cdots {\bar{\nu }}_q} \beta ^{\mu _1\cdots \mu _p {\bar{\nu }}_1\cdots {\bar{\nu }}_q} (\star 1 ) = \alpha \star (\beta )^*. \end{aligned}$$

It has a complex volume form, which is nowhere vanishing and globally well defined:

$$\begin{aligned} \Omega = \frac{1}{3!} \Omega _{\mu \nu \rho } \text {d}x^\mu \text {d}x^\nu \text {d}x^\rho , \qquad ||\Omega ||^2 = \frac{1}{3!} \Omega _{\mu \nu \rho } {\overline{ \Omega }}^{\mu \nu \rho }, \end{aligned}$$

where $||\Omega ||$ is a coordinate scalar, and so a constant for a fixed manifold, but depends on parameters, denoted *y*. We can write

A.3 $$\begin{aligned} \Omega _{\mu \nu \rho } = f(x,y) \epsilon _{\mu \nu \rho }, \qquad \epsilon _{123} = 1, \end{aligned}$$

where $e_{\mu \nu \rho }$ is the permutation symbol and *f*(*x*, *y*) is a holomorphic function of coordinates and parameters. $\epsilon _{\mu \nu \rho }$ is not a tensor and consequently *f* transforms like $g^{1/4}$ under holomorphisms (holomorphic diffeomorphisms): if $x' = x'(x)$, then *f*(*x*) transforms $f'(x) = (\det j) f(x)$, where $j^\mu _\nu = \frac{\partial x'{}^\mu }{\partial x^\nu }$. It satisfies the relation

A.4 $$\begin{aligned} |f|^2 = g^{1/2} ||\Omega ||^2, \end{aligned}$$

The complex volume form $\Omega$ transform like sections of a complex line bundle on . Under a gauge transformation $\Omega \rightarrow \mu \Omega$ with , we have $||\Omega ||^2 \rightarrow |\mu |^2 ||\Omega ||^2$ while *g* is invariant. It is sometimes convenient to isolate the phases of *f* and $\mu$:

$$\begin{aligned} f = |f| e^{\text {i}\zeta }, \qquad \mu = |\mu | e^{\text {i}\xi }, \end{aligned}$$

If *A*, *B*, *C* are (0, 1)-forms, then

A.5 $$\begin{aligned} A_{\bar{\mu }}\,B_{\bar{\nu }}\, C_{\overline{\rho }}\,\, \Omega ^{{\bar{\mu }}{\bar{\nu }}{\overline{\rho }}} \star 1 = \text {i}\, \Omega \, \,A B C, \qquad A B C = \frac{1}{||\Omega ||^2}\, A_{\bar{\mu }}\, B_{\bar{\nu }}\, C_{\overline{\rho }}\,\,\Omega ^{{\bar{\mu }}{\bar{\nu }}{\overline{\rho }}}\,\, {\overline{ \Omega }}.\qquad \end{aligned}$$

where we have the compatibility relation

$$\begin{aligned} \frac{\text {i}\Omega \, {\overline{ \Omega }}}{||\Omega ||^2} = \frac{1}{3!} \,\omega ^3 = \text {d}^6x g^\frac{1}{2}. \end{aligned}$$

It is also useful to note

$$\begin{aligned} \,{\overline{ \star }\,}\Omega = \text {i}\Omega , \qquad \Omega \,{\overline{ \star }\,}\Omega = {||\Omega ||}^2 \star 1 = \text {i}\Omega {\overline{ \Omega }}. \end{aligned}$$

In coordinates

A.6 $$\begin{aligned} \star 1 =\frac{\sqrt{g}}{(3!)^2} \text {i}\epsilon _{\mu _1\mu _2 \mu _3} \epsilon _{{\bar{\nu }}_1{\bar{\nu }}_2 {\bar{\nu }}_3} \text {d}x^{\mu _1} \text {d}x^{\mu _2}\text {d}x^{\mu _3} \text {d}x^{{\bar{\nu }}_1}\text {d}x^{{\bar{\nu }}_2} \text {d}x^{{\bar{\nu }}_3}. \end{aligned}$$

## B Spinors

We establish some conventions and results for spinors in $d=4$, $d=6$ and $d=10$. We define the Pauli matrices as

B.1 $$\begin{aligned} \begin{aligned} \sigma ^1&= \begin{pmatrix} 0 &{} \quad 1\\ 1 &{} \quad 0 \end{pmatrix}, \qquad \sigma ^2 = \begin{pmatrix} 0 &{} \quad -\text {i}\\ \text {i}&{} \quad 0 \end{pmatrix}, \qquad \sigma ^3 = \begin{pmatrix} 1 &{} \quad 0\\ 0 &{} \quad -1 \end{pmatrix}, \\\end{aligned} \end{aligned}$$

and we denote ${\mathbb {1}}_n$ the $n\times n$ identity matrix. We also define

$$\begin{aligned} \sigma ^0 = \begin{pmatrix} -1 &{} \quad 0 \\ 0 &{} \quad -1 \end{pmatrix}. \end{aligned}$$

We denote $\sigma ^{e f} = \frac{1}{2}(\sigma ^{e} \sigma ^{f} - \sigma ^{f} \sigma ^{e})$. A similar definition applies for the gamma matrices.

### B.1 Spinors in flat space

#### B.1.1 ${\mathfrak {so}}(9,1)$

The Dirac representation of ${\mathfrak {so}}(9,1)$ is 32-dimensional. We denote the 32-dimensional ${\mathfrak {so}}(9,1)$ gamma matrices  and chirality operator . The Dirac spinor decomposes into two Majorana–Weyl representations ${{\varvec{32}}} = {{\varvec{16}}}\oplus {{\varvec{16}}}'$. Our notation will be that primed representations are negative chirality spinors; unprimed representations are positive chirality spinors.

Let $\varepsilon$ be Weyl spinor that is of positive chirality . As  and  both satisfy the same Lorentz algebra as , there are two similarity transformations preserving the Lorentz algebra

B.2

under which the spinor transforms to $\varepsilon \rightarrow B_i \varepsilon$. Hence, $\varepsilon ^*$ and $B_i \varepsilon$ transform in the same way under Lorentz transformations, and we can define Majorana conjugation to be

$$\begin{aligned} \varepsilon ^c = B_{(i)}^{-1} \varepsilon ^*, \end{aligned}$$

and the Majorana condition is $\varepsilon = \varepsilon ^c$. Applying Majorana conjugation twice gives a consistency condition $B_i^* B_i = 1$, no sum on the *i*, which must be satisfied. For ${\mathfrak {so}}(9,1)$ it is possible to find both $B_{(1)}$ and $B_{(2)}$ satisfying (B.2) that also satisfy the consistency condition; this is not true for ${\mathfrak {so}}(3,1)$ and ${\mathfrak {so}}(6)$. In the text we utilise $B_{(2)}$, which, with our choice of basis, gives a manifestly consistent Majorana condition for ${\mathfrak {so}}(3,1)\oplus {\mathfrak {so}}(6)$.

We utilise the convention of complex conjugation of a pair of spinors interchanging their order without introducing a sign.

#### B.1.2 ${\mathfrak {so}}(3,1)$

We work with mostly positive signature. A basis of Dirac gamma matrices are

B.3 $$\begin{aligned} \gamma ^e = \begin{pmatrix} 0&{} \quad \sigma ^e\\ -{\overline{\sigma }}^e &{} \quad 0 \end{pmatrix}, \end{aligned}$$

where $\sigma ^e = (\sigma ^0, \sigma ^1, \sigma ^2, \sigma ^3)$ with $\sigma ^0= -{\mathbb {1}}_2$, and the remaining matrices are the Pauli matrices (B.1). The conjugate matrices ${\overline{\sigma }}^0 = (\sigma ^0, -\sigma ^1, -\sigma ^2, -\sigma ^3)$. $\gamma ^0$ is anti-Hermitian $(\gamma ^0)^2 = -{\mathbb {1}}_4$, while $\gamma ^1,\ldots ,\gamma ^3$ are all Hermitian. Complex conjugation is the same as transpose: $(\sigma ^e)^* = (\sigma ^e)^t$. We denote $\gamma ^{ef} = \frac{1}{2}(\gamma ^e \gamma ^f - \gamma ^f \gamma ^e)$.

The chirality matrix is

$$\begin{aligned} \gamma ^{(4)} = -\text {i}\gamma ^0 \cdots \gamma ^3 = \begin{pmatrix} -{\mathbb {1}}_2 &{} \quad 0 \\ 0 &{} \quad {\mathbb {1}}_2 \end{pmatrix}. \end{aligned}$$

The Majorana conjugate of a Dirac spinor $\Psi$ is

B.4 $$\begin{aligned} \Psi ^c = B_4^{-1} \Psi ^*,\qquad B_4 = \begin{pmatrix} 0 &{} \quad -\varepsilon \\ \varepsilon &{} \quad 0 \end{pmatrix}, \end{aligned}$$

where $\varepsilon = \text {i}\sigma ^2$. It can be checked that $B_4 \gamma ^e B_4^{-1} = (\gamma ^e)^*$ and that $B_4 B_4^* = 1$ so that $(\Psi ^c)^c = \Psi$.

The ${{\varvec{4}}}$ of ${\mathfrak {so}}(3,1)$ admits a pair of Weyl representations

$$\begin{aligned} {{\varvec{4}}} = {{\varvec{2}}} \oplus {{\varvec{2}}}', \qquad \qquad \Psi = \begin{pmatrix} 0 \\ \zeta \end{pmatrix} + \begin{pmatrix} \zeta ' \\ 0 \end{pmatrix}. \end{aligned}$$

of positive and negative chirality, respectively, and in the second equality, expressed as spinors in the basis (B.3). This is sometimes denoted $\Psi = \zeta \oplus \zeta '$.

Where possible, we adopt the 2-component spinor notation, see, for example, [31, 32]. The indices on Weyl spinors are denoted by ${\dot{a}}$ and *a*. The rule for raising and lowering is through the $\epsilon$ permutation symbol where $\epsilon ^{12} = \epsilon _{21} = 1$ and $\epsilon ^{21} = \epsilon _{12} = -1$:

B.5 $$\begin{aligned} \begin{aligned} \zeta '^a&= \epsilon ^{ab} \zeta '_b, \qquad \zeta '_a = \epsilon _{ab} \zeta '^b,\\ {\overline{\zeta }}^{\dot{a}}&= \epsilon ^{{\dot{a}}{\dot{b}}} {\overline{\zeta }}_{\dot{b}}, \qquad {\overline{\zeta }}_{\dot{a}}= \epsilon _{{\dot{a}}{\dot{b}}} {\overline{\zeta }}^{\dot{b}}. \end{aligned} \end{aligned}$$

Complex conjugation exchanges dots on indices $(\zeta ^a)^* = {\overline{\zeta }}^{\dot{a}}$, $(\epsilon _{ab})^* = \epsilon _{{\dot{a}}{\dot{b}}}$, etc. These spinors are assigned the Grassmann odd property and so anticommute. However, when complex conjugating a pair of spinors, the order is interchanged without a sign: $(\zeta _a \zeta '_b)^* = {\overline{\zeta }}'_{\dot{b}}{\overline{\zeta }}_{\dot{a}}$.

The indices on $\sigma ^e$ and ${\overline{\sigma }}^e$ are related

$$\begin{aligned} \sigma ^e_{a{\dot{a}}} = \epsilon _{a b} {\overline{\sigma }}^{f\,{\dot{b}}b} \epsilon _{{\dot{a}}{\dot{b}}}, \end{aligned}$$

and the index structure on $\Psi$ is

B.6 $$\begin{aligned} \Psi = \begin{pmatrix} \zeta '_a \\ {\overline{\zeta }}^{\dot{a}}\end{pmatrix}. \end{aligned}$$

When indices are not written there is an implicit contraction through the $\epsilon$ symbol. For example,

B.7 $$\begin{aligned} \zeta \zeta ' = \zeta ^a \zeta '_a = \epsilon ^{ab}\zeta _b \zeta '_a = - \epsilon ^{ab} \zeta '_a \zeta _b = \zeta '{}^b \zeta _b = \zeta ' \zeta . \end{aligned}$$

Analogous conventions exist for dotted indices, given by complex conjugating the above equation. Some useful spinor relations to be used inside actions are:

B.8 $$\begin{aligned} \begin{aligned} \zeta ' \sigma ^e \partial _e {\overline{\zeta }}= {\overline{\zeta }}{\overline{\sigma }}^e \partial _e \zeta ', \qquad (-\text {i}\zeta ' \sigma ^e \partial _e {\overline{\zeta }})^* = - \text {i}\zeta \sigma ^e \partial _e {\overline{\zeta }}' = - \text {i}{\overline{\zeta }}' {\overline{\sigma }}^e \partial _e \zeta . \end{aligned} \end{aligned}$$

The Dirac conjugate of a Dirac spinor is

$$\begin{aligned} {\overline{\Psi }}= \Psi ^\dag \gamma ^0. \end{aligned}$$

A slight abuse of notation: the bar on a Dirac spinor denotes the Dirac conjugate, while the bar on a Weyl spinor denotes a dotted index.

The kinetic term for the Dirac spinor in terms of Weyl spinors (B.6)

B.9 $$\begin{aligned} \text {i}{\overline{\Psi }}\gamma ^e \partial _e \Psi = \text {i}{\overline{\zeta }}' {\overline{\sigma }}^e \partial _e \zeta ' + \text {i}\zeta \sigma ^e\partial _e {\overline{\zeta }}'. \end{aligned}$$

A Lorentz transformation on a Dirac spinor is $\delta \Psi = \Lambda _{ef} \gamma ^{ef}$, with $\Lambda _{ef} = - \Lambda _{fe}$ and in the basis (B.3) becomes an action on Weyl spinors

B.10 $$\begin{aligned} \begin{pmatrix} \delta \zeta _a' \\ \delta {\overline{\zeta }}^{\dot{a}}\end{pmatrix} = \Lambda _{ef} \begin{pmatrix} - (\sigma ^{e} {\overline{\sigma }}^{f})_a{}^b &{} \quad 0 \\ 0 &{} \quad - ({\overline{\sigma }}^{e} \sigma ^{f})^{\dot{a}}{}_{\dot{b}}\end{pmatrix} \begin{pmatrix} \zeta _b' \\ {\overline{\zeta }}^{\dot{b}}\end{pmatrix}, \end{aligned}$$

from which we identify the transformation properties of $\zeta _a'$ and ${\overline{\zeta }}^{\dot{a}}$, and identify these with the ${{\varvec{2}}}'$ and ${{\varvec{2}}}$ of ${\mathfrak {so}}(3,1)$, respectively.

The Majorana conjugate is

B.11 $$\begin{aligned} \Psi = \begin{pmatrix} \zeta '_a \\ {\overline{\zeta }}^{\dot{a}}\end{pmatrix} \qquad \qquad \Psi ^c = B^{-1} \Psi ^* = \begin{pmatrix} \zeta _a\\ {\overline{\zeta }}'^{\dot{a}}\end{pmatrix} \end{aligned}$$

and as expected $\Psi$ and $\Psi ^c$ have the same index structure, with Majorana conjugation swapping the prime, reflecting the fact they transform in the same way under Lorentz transformations. Notice that complex conjugation of a Weyl spinor does not by itself give another Weyl spinor. For example, $\zeta _a$ transforms as the ${{\varvec{2}}}'$ but ${\overline{\zeta }}_{\dot{a}}$ does not transform as a ${{\varvec{2}}}$, as can be seen by conjugating the top line of (B.10) and comparing with the second line. Instead, one is to complex conjugate and contract with $\epsilon$: $(\epsilon ^{ab} \zeta _b)^* = \epsilon ^{{\dot{a}}{\dot{b}}} {\overline{\zeta }}_{\dot{b}}$ transforms in the ${{\varvec{2}}}$.

In this basis, a Majorana spinor satisfies $\zeta _a= \zeta _a'$. We will not impose this and choose to work in the Weyl basis.

#### B.1.3 ${\mathfrak {so}}(6)$

We describe ${\mathfrak {so}}(6)$ spinors first in flat space, before coupling them to a curved manifold with ${\mathfrak {su}}(3)$-structure in the next section.

A Dirac spinor decomposes into a pair of Weyl representations

$$\begin{aligned} {{\varvec{8}}} = {{\varvec{4}}} \oplus {{\varvec{4}}}'. \end{aligned}$$

A basis compatible with this is

B.12 $$\begin{aligned} \begin{aligned} \gamma ^1&= \sigma ^1 \otimes \sigma ^3 \otimes \sigma ^3, \quad \gamma ^2 = \sigma ^2 \otimes \sigma ^3 \otimes \sigma ^3, \quad \gamma ^3 = {\mathbb {1}}_2 \otimes \sigma ^1 \otimes \sigma ^3, \\\gamma ^4&= {\mathbb {1}}_2\otimes \sigma ^2 \otimes \sigma ^3, \, \quad \gamma ^5 = {\mathbb {1}}_2 \otimes {\mathbb {1}}_2 \otimes \sigma ^1, \quad \gamma ^6 = {\mathbb {1}}_2 \otimes {\mathbb {1}}_2 \otimes \sigma ^2.\\ \end{aligned} \end{aligned}$$

The chirality operator is

B.13 $$\begin{aligned} \gamma ^{(6)} = \text {i}\gamma ^1 \ldots \gamma ^6 = \sigma ^3 \otimes \sigma ^3 \otimes \sigma ^3. \end{aligned}$$

We define raising and lowering operators, introducing holomorphic and antiholomorphic indices

$$\begin{aligned} \gamma ^\mu = \frac{1}{2}(\gamma ^{2\mu -1} + \text {i}\gamma ^{2\mu }), \qquad \gamma ^{\bar{\mu }}= \frac{1}{2}(\gamma ^{2{\bar{\mu }}-1} - \text {i}\gamma ^{2{\bar{\mu }}}). \end{aligned}$$

These are real $(\gamma ^\mu )^* = \gamma ^\mu$, related by $(\gamma ^\mu )^\dag = \gamma ^{\bar{\mu }}$ and satisfy $\{ \gamma ^\mu , \gamma ^{\bar{\nu }}\} = \delta ^{\mu {\bar{\nu }}}$. In this basis only ${\mathfrak {so}}(2)\oplus {\mathfrak {so}}(2)\oplus {\mathfrak {so}}(2)\subset$ is manifestly preserved.

The conjugation matrix $B_6$ satisfies

$$\begin{aligned} B_6 \gamma ^m B_6^{-1} = - (\gamma ^m)^*, \end{aligned}$$

and in this basis is the product of all imaginary matrices so that

B.14 $$\begin{aligned} B_6 = \gamma ^2 \gamma ^4 \gamma ^6 = \sigma ^2 \otimes -\text {i}\sigma ^1 \otimes \sigma ^2 = \text {i}\prod _{\mu =1}^3 (\gamma ^\mu - \gamma ^{\bar{\mu }}), \end{aligned}$$

which satisfies $$ B_6^{-1} {=} B_6^* {=} -B_6$$ and so an $${\mathfrak {so}}(6)$$ spinor $$\lambda $$ satisfies $$(\lambda ^c)^c = \lambda $$. In the second equality, we have written $$B_6$$ in terms of raising and lowering operators. The conjugation matrix changes chirality $$B_6 \gamma ^\mu _\pm {=} \gamma ^\mu _\mp B_6$$.

### B.2 Spinors on

In discussing , we take the 32-dimensional gamma matrices to decompose

where $\gamma ^e$ are four-dimensional matrices in (B.3) and $\gamma ^m$ are the 8-dimensional matrices in (B.12). The chirality matrix is

The complex conjugation matrix

B.15 $$\begin{aligned} B = B_4 \otimes B_6, \end{aligned}$$

where $B_4$ is in (B.4) and $B_6$ is in (B.14). *B* is imaginary, unitary and anti-Hermitian $B^\dag = B^{-1} = -B$ and satisfies the property that

The Majorana conjugate of a spinor $\varepsilon$ in the ${{\varvec{32}}}$ is $\varepsilon ^c = B^{-1} \varepsilon ^*.$ Expand $\varepsilon$ in terms of ${\mathfrak {so}}(3,1)\oplus {\mathfrak {so}}(6)$ spinors

$$\begin{aligned} \varepsilon = \zeta \otimes \lambda \oplus \zeta ' \otimes \lambda ' ~\cong ~ \begin{pmatrix} 0\\ {\overline{\zeta }}^{\dot{a}}\end{pmatrix}\otimes \lambda + \begin{pmatrix} \zeta _a'\\ 0 \end{pmatrix}\otimes \lambda ', \end{aligned}$$

where $\lambda$ and $\lambda '$ are in the ${{\varvec{4}}}$ and ${{\varvec{4}}}'$, respectively, while $\zeta$ and $\zeta '$ are in the ${{\varvec{2}}}$ and ${{\varvec{2}}}'$, respectively. In the second line we have written this in terms of the four-dimensional ${\mathfrak {so}}(3,1)$ spinors, with their spinor indices explicit; we will always leave the ${\mathfrak {so}}(6)$ spinor indices implicit.

The Majorana condition $\varepsilon ^c = \varepsilon$ is simplified by using (B.11),

B.16 $$\begin{aligned} \begin{aligned} \begin{pmatrix} 0 \\ {\overline{\zeta }}^{\dot{a}}\end{pmatrix} \otimes \lambda = \begin{pmatrix} 0 \\ {\overline{\zeta }}'{}^{\dot{a}}\end{pmatrix} \otimes \lambda '{}^c, \qquad \begin{pmatrix} \zeta _a' \\ 0 \end{pmatrix} \otimes \lambda '&= \begin{pmatrix} \zeta _a \\ 0 \end{pmatrix} \otimes \lambda ^c. \end{aligned} \end{aligned}$$

It is sometimes more convenient to write this simply as

B.17 $$\begin{aligned} {\overline{\zeta }}^{\dot{a}}\otimes \lambda = {\overline{\zeta }}'{}^{\dot{a}}\otimes \lambda '^c. \end{aligned}$$

The Majorana–Weyl spinor $\varepsilon$ can now be written solely in terms of, for example, $\zeta ',\lambda '$:

$$\begin{aligned} \varepsilon = \begin{pmatrix} 0\\ {\overline{\zeta }}'^{\dot{a}}\end{pmatrix}\otimes \lambda '{}^c + \begin{pmatrix} \zeta _a'\\ 0 \end{pmatrix}\otimes \lambda ', \end{aligned}$$

### B.3 Spinors on a complex manifold with ${\mathfrak {su}}(3)$-structure

The manifold  is endowed with an ${\mathfrak {su}}(3)$-structure meaning that there is a globally well-defined non-vanishing spinor implying a reduction of the structure group ${\mathfrak {so}}(6) \rightarrow {\mathfrak {su}}(3)$ under which

B.18 $$\begin{aligned} {{\varvec{4}}} = {{\varvec{3}}} \oplus {{\varvec{1}}}, \qquad {{\varvec{4}}}' = {{\overline{\varvec{3}}}} \oplus {{\varvec{1}}}, \end{aligned}$$

and the spinors decompose, respectively, as

$$\begin{aligned} \lambda = \lambda _{{{\varvec{3}}}} \oplus \lambda _+,\qquad \qquad \lambda ' = \lambda _{{{\overline{\varvec{3}}}}} \oplus \lambda _-. \end{aligned}$$

The spinors $\lambda _+, \lambda _-$ are the ${\mathfrak {su}}(3)$ invariant spinors that are nowhere vanishing on the manifold  that define the ${\mathfrak {su}}(3)$-structure.

With respect to the basis (B.12), the raising and lowering matrices $\gamma _+^\mu$ and $\gamma _-^{\bar{\nu }}$ are real and related by Hermitian conjugation $(\gamma _+^\mu )^\dag = \gamma _-^{\bar{\mu }}$. This reality property is consequence of our choice of basis, and any physical result will not depend on this choice. Care must be taken when interpreting the holomorphy of indices, and where any ambiguity may arise, we will keep the ± subscript. Nonetheless, at the end of a calculation we will be able to interpret the indices in terms of holomorphic or antiholomorphic indices of .

The matrices satisfy an algebra

$$\begin{aligned} \{ \gamma ^\mu , \gamma ^{\bar{\nu }}\} = g^{\mu {\bar{\nu }}}, \end{aligned}$$

where $\mu ,{\bar{\nu }}$ are coordinate indices and the right-hand side is the inverse metric.^9^

Majorana conjugation is defined using a covariant version of *B* in (B.14). On an ${\mathfrak {su}}(3)$ manifold, *B* is a coordinate scalar, gauge invariant, and satisfies the property that

$$\begin{aligned} B \gamma ^m B^{-1} = - (\gamma ^m)^*, \qquad B^* B = 1, \quad B^\dag = B^{-1}. \end{aligned}$$

This fixes

B.19 $$\begin{aligned} B = \text {i}g^{1/4} \left( \frac{1}{3!} \epsilon _{\mu \nu \rho } \gamma _+^{\mu \nu \rho } + \frac{1}{2} \epsilon _{\mu \nu \rho } \gamma _+^\mu \gamma _-^{\nu \rho } -\frac{1}{2} \epsilon _{\mu \nu \rho } \gamma _+^{\mu \nu } \gamma _-^\rho - \frac{1}{3!} \epsilon _{{\mu \nu \rho }} \gamma _-^{{\mu \nu \rho }}\right) \qquad \end{aligned}$$

This is the main example where confusion can arise in holomorphy of indices, and so we use the ± subscript for clarity.

We build spinor representations by lowering and raising operators. Denote the lowest weight state $\lambda _-$, satisfying $\gamma ^{\bar{\mu }}\lambda _- = 0$. We define the remaining spinors as follows:

B.20 $$\begin{aligned} \begin{aligned} \lambda _-&\qquad \qquad {{\varvec{1}}},\\ \lambda _{{{\varvec{3}}}}:= \Lambda _\mu \gamma ^\mu \lambda _-&\qquad \qquad {{\varvec{3}}},\\ \lambda _{{{\overline{\varvec{3}}}}} ~:=~ \frac{1}{2}\Lambda _{\mu \nu } \gamma ^{\mu \nu } \lambda _-&\qquad \qquad {{\overline{\varvec{3}}}},\\ \lambda _+:= \frac{1}{3!} \Lambda _+ \epsilon _{\mu \nu \rho }\gamma ^{\mu \nu \rho } \lambda _-&\qquad \qquad {{\varvec{1}}}. \end{aligned} \end{aligned}$$

where $\gamma ^{\mu \nu } = \frac{1}{2}(\gamma ^\mu \gamma ^\nu - \gamma ^\nu \gamma ^\mu )$ and . Note that $\gamma ^{\mu \nu } \lambda _- = \gamma ^\mu \gamma ^\nu \lambda _-$. Here $\epsilon _{\mu \nu \rho }$ is the permutation symbol with $\epsilon _{123} = 1$ and $\Lambda _+$ is a tensor density to be fixed.

To identify $\Lambda _+$ we study its transformation properties under symmetries of the moduli space and under holomorphisms. First note that $\Lambda _+$ transforms like $g^{1/4}$ under holomorphisms. Hence, $\Lambda _+ \propto g^{1/4}$ up to a parameter-dependent coordinate scalar. Second, recall the gauge symmetry $\Omega \rightarrow \mu \Omega$ where . Under this symmetry the fermions $\lambda _\pm$ are charged transforming under the  as^10^

B.21 $$\begin{aligned} \lambda _\pm \rightarrow \lambda _\pm e^{\text {i}\xi /2}, \end{aligned}$$

and so $\Lambda _+$ transforms as $\Lambda _+ \rightarrow \Lambda _+ e^{\text {i}\xi }$. Hence, $\Lambda _+ \propto (f \sqrt{g}) / |f|$, now fixed up to a gauge-neutral coordinate scalar. If we demand that $\lambda _+^\dag \lambda _+ = \lambda _-^\dag \lambda _-$ this fixes the constant to be a phase and we can write the final result as

B.22 $$\begin{aligned} \Lambda _+ = e^{\text {i}\phi } \frac{f }{||\Omega ||}, \end{aligned}$$

for some phase $e^{\text {i}\phi }$. There are three phases of interest: $\psi _\pm = \arg \lambda _\pm$ and $\zeta$ the phase of $f = |f| e^{\text {i}\zeta }$. The gauge symmetry eliminates one of these degrees of freedom, and we can form two gauge-invariant combinations $\phi = \psi _+ - \psi _- - \zeta$ and $\Psi = \psi _+ + \psi _-$.

Using one of these global symmetries we could choose $\phi = 0$, which in the gauge where $\zeta = 0$ amounts to fixing the relative phases of $\lambda _\pm$ equal.

We state the final result as

B.23 $$\begin{aligned} \lambda _+ = \frac{e^{\text {i}\phi } }{||\Omega ||} \frac{1}{3!} \Omega _{\mu \nu \rho } \gamma ^{\mu \nu \rho } \lambda _-, \end{aligned}$$

The norms $\lambda _\pm ^\dag \lambda _\pm$ are gauge-invariant coordinate scalars, and so we are free to fix them to be unity

B.24 $$\begin{aligned} \lambda _\pm ^\dag \lambda _\pm = 1. \end{aligned}$$

We note that $\lambda _{{\overline{\varvec{3}}}}$ can be written as

B.25 $$\begin{aligned} \lambda _{{{\overline{\varvec{3}}}}} = \Lambda '_{\bar{\mu }}\gamma ^{\bar{\mu }}\lambda _+, \quad \Lambda _{\bar{\mu }}' = \frac{e^{-\text {i}\phi }}{2||\Omega ||} {\overline{ \Omega }}_{\overline{\mu \nu \rho }}\Lambda ^{\overline{\nu \rho }}, \quad \Lambda _{\mu \nu } = \frac{e^{\text {i}\phi }}{||\Omega ||} \Omega _{\mu \nu }{}^{\overline{\rho }}\Lambda _{\overline{\rho }}'. \end{aligned}$$

By studying $\lambda _+^\dag \lambda _+$ in two different ways we identify

B.26 $$\begin{aligned} \Omega _{\mu \nu \rho }= - e^{-\text {i}\phi }\,||\Omega ||\, \lambda _-^\dag \gamma _{\mu \nu \rho } \lambda _+,\qquad {\overline{ \Omega }}_{\overline{\mu \nu \rho }}= e^{\text {i}\phi }\,||\Omega ||\, \lambda _+^\dag \gamma _{\overline{\mu \nu \rho }} \lambda _-, \end{aligned}$$

as well as

B.27 $$\begin{aligned} \lambda _+^\dag \gamma ^\mu \gamma ^{\bar{\nu }}\lambda _+ = g^{\mu {\bar{\nu }}}, \qquad \lambda _-^\dag \gamma ^{\bar{\nu }}\gamma ^\mu \lambda _- = g^{\mu {\bar{\nu }}}. \end{aligned}$$

The norms of spinors are then

B.28 $$\begin{aligned} \lambda _{{{\varvec{3}}}}^\dag \lambda _{{{\varvec{3}}}} = \Lambda _\mu \Lambda ^\mu , \qquad \lambda _{{{\overline{\varvec{3}}}}}^\dag \lambda _{{{\overline{\varvec{3}}}}} = \Lambda _{\bar{\mu }}' \Lambda ^{\bar{\mu }}= \frac{1}{2}\Lambda _{\mu \nu }\Lambda ^{\mu \nu }. \end{aligned}$$

It is useful to tabulate Majorana conjugates of spinors $\lambda ^c = B^{-1} \lambda ^* = (B\lambda )^*$:

B.29 $$\begin{aligned} \begin{aligned} \lambda _-^c&= -\text {i}g^{1/4} \Lambda _+^{*\,-1} \lambda _+^* =-\text {i}\lambda _+, \\ \lambda _+^c&= -\text {i}g^{-1/4} \Lambda _+^* \lambda _-^* = -\text {i}\lambda _-,\\ \lambda _{{{\overline{\varvec{3}}}}}^c&= \text {i}g^{-1/4}\Lambda _+^* \Lambda '{}_\mu ^* \gamma ^\mu \lambda _-^* = \text {i}\Lambda '{}_\mu ^* \gamma ^\mu \lambda _-, \\ \lambda _{{{\varvec{3}}}}^c&= \text {i}g^{1/4}\Lambda _+^{*\,-1} \Lambda {}_{\bar{\mu }}^* \gamma ^{\bar{\mu }}\lambda _+^* = \text {i}\Lambda _{\bar{\mu }}^* \gamma ^{\bar{\mu }}\lambda _+, \end{aligned} \end{aligned}$$

and

B.30 $$\begin{aligned} (\lambda _{{{\overline{\varvec{3}}}}}^c)^{\dag } = -\text {i}\Lambda '{}_{\bar{\mu }}\lambda _-^\dag \gamma ^{\bar{\mu }}, \qquad (\lambda _{{{\varvec{3}}}}^c)^{\dag } = - \text {i}\Lambda _\mu \lambda _+^\dag \gamma ^\mu . \end{aligned}$$

Finally, given a derivative operator $D_{\bar{\nu }}$, which in the text becomes a covariant derivative with respect to the bundle symmetries, we will need the following bilinear

B.31 $$\begin{aligned}&\left( \lambda _{{{\overline{\varvec{3}}}}}^c\right) ^{\dag } \gamma ^{\bar{\nu }}D_{\bar{\nu }}\lambda _{{{\overline{\varvec{3}}}}} = \text {i}e^{\text {i}\phi }\,\Big ( \Lambda _{\bar{\mu }}' D_{\bar{\nu }}\Lambda _{\overline{\rho }}' \Big ) \,\frac{ \Omega ^{\overline{\mu \nu \rho }} }{{||\Omega ||}}, \quad (\lambda _{{{\varvec{3}}}}^c)^{\dag } \gamma ^\nu D_\nu \lambda _{{{\varvec{3}}}}\nonumber \\&\quad = -\text {i}e^{-\text {i}\phi }\,\Big ( \Lambda _\mu D_\nu \Lambda _\rho \Big ) \,\frac{ {\overline{ \Omega }}^{\mu \nu \rho } }{{||\Omega ||}}. \end{aligned}$$

### B.4 Spinors charged under gauge symmetries

Sometimes the spinors carry additional structure, for example being charged in a representation of ${\mathfrak {g}}\oplus {\mathfrak {h}}$. In that case complex conjugation is promoted to Hermitian conjugation.

Consider first $\zeta ,\zeta '$; these spinors may be charged in a representation of ${\mathfrak {g}}$. In computing a Majorana conjugate, the complex conjugate in (B.11) is promoted to Hermitian conjugate on the gauge structure. It is normally easy to do this with the spinor indices explicit; Majorana conjugation does not transpose the spinor structure.

As a way of illustration, there are three relevant cases to the text. The first are when $\zeta ,\zeta '$ are singlets under $\mathrm{ad }_{\mathfrak {g}}$, in which Majorana conjugation is unchanged from the previous subsection. The second case, $\zeta$ is in a representation ${{\varvec{R}}}$, denoted $\zeta '_{{{\varvec{R'}}}}$, and Hermitian conjugation acts as $(\zeta _{{\varvec{R}}}^a)^\dag ~\cong ~ {\overline{\zeta }}^{\dot{a}}_{{{\overline{\varvec{R}}}}}$. The third case is when $\zeta '$ is in the adjoint of ${\mathfrak {g}}$, in which it is anti-Hermitian $(\zeta _{\mathrm{ad }_{\mathfrak {g}}}^a)^\dag =- {\overline{\zeta }}^{\dot{a}}_{\mathrm{ad }_{\mathfrak {g}}}$. The Majorana conjugates of the last two cases are explicitly:

B.32 $$\begin{aligned} \begin{pmatrix} \zeta '_{{{\varvec{R'}}}\,a} \\ 0 \end{pmatrix}^c = B_4^{-1} \begin{pmatrix} (\zeta '_{{{\varvec{R}}}'\,a})^\dag \\ 0 \end{pmatrix} = \begin{pmatrix} 0 \\ {\overline{\zeta }}'{}^{\dot{a}}_{{\overline{\varvec{R'}}}} \end{pmatrix},\qquad \begin{pmatrix} \zeta '_{\mathrm{ad }_{\mathfrak {g}}\,a} \\ 0 \end{pmatrix}^c ~=- \begin{pmatrix} 0 \\ {\overline{\zeta }}'{}^{\dot{a}}{} \end{pmatrix}. \end{aligned}$$

The Majorana condition (B.17) implies that if $\zeta '$ is in the ${{\varvec{R}}}$, then $\zeta$ is in the ${{\overline{\varvec{R}}}}$. Of course, if the representation is real, then ${{\varvec{R}}} = {{\overline{\varvec{R}}}}$ in the above.

Similar comments apply when $\lambda ,\lambda '$ carry representations of ${\mathfrak {h}}$. Only $\lambda _{{{\varvec{3}}}}$ and $\lambda _{{{\overline{\varvec{3}}}}}$ turn out to carry non-trivial representations of ${\mathfrak {h}}$, and this is through the object $\Lambda _\mu$ and $\Lambda '_{\bar{\mu }}$ in (B.20) and (B.25). The singlets $\lambda _\pm$ are always gauge singlets. The generalisation of (B.29) is

B.33 $$\begin{aligned} \begin{aligned} \lambda _\pm ^c&= -\text {i}\lambda _\mp , \qquad \lambda _{{{\overline{\varvec{3}}}}}^c = \text {i}\Lambda '{}_\mu ^\dag \gamma ^\mu \lambda _-, \qquad \lambda _{{{\varvec{3}}}}^c = \text {i}\Lambda _{\bar{\mu }}^\dag \gamma ^{\bar{\mu }}\lambda _+, \end{aligned} \end{aligned}$$

where $\Lambda _\mu$ is charged in a representation and $\Lambda _{\bar{\mu }}^\dag$ is the appropriate Hermitian conjugate.

Putting this into (B.17) determines $\zeta \otimes \lambda$ in terms of $\zeta '\otimes \lambda '$:

B.34 $$\begin{aligned} {\overline{\zeta }}^{\dot{a}}\otimes \lambda _+ ~= -\text {i}{\overline{\zeta }}'{}^{{\dot{a}}\,\dag } \otimes \lambda _+, \quad {\overline{\zeta }}^{\dot{a}}\otimes \Lambda _\mu \gamma ^\mu \lambda _- =\text {i}{\overline{\zeta }}'{}^{{\dot{a}}\,\dag } \otimes \Lambda '{}^\dag _\mu \gamma ^\mu \lambda _-. \end{aligned}$$

### B.5 Some useful spinor bilinears

We express the Majorana–Weyl spinor $\varepsilon$ in terms of $\zeta ',\lambda '$ and list some bilinears relevant to the main text.

B.35

We have left the four-dimensional spinor indices for clarity, but will now drop them in spinor contractions, using convention (B.7).

We can now evaluate these relations for some specific examples relevant to the text when the spinors are charged in representations of ${\mathfrak {g}}\oplus {\mathfrak {h}}$:

1. of ${\mathfrak {g}}\oplus {\mathfrak {h}}$. Use $(\zeta '{}^a)^\dag = - {\overline{\zeta }}'^{{\dot{a}}}$, its Majorana conjugate (B.32), as well as $\lambda '=\lambda _-$ and $\lambda '^c = -\text {i}\lambda _+$:
  B.36
  where in the last line $\lambda _+^\dag \gamma ^\mu \lambda _- = 0$. In the third line we understand this will appear integrated and so use integration by parts ${\overline{\zeta }}'\, {\overline{\sigma }}^{e} \,\partial _e\, \zeta ' = \zeta ' \,\sigma ^e \,\partial _e \,{\overline{\zeta }}'$.
2. $$\zeta '\otimes \lambda _{{{\overline{\varvec{3}}}}} \in ({{\varvec{R_i}}}, {{\overline{\varvec{r_i}}}})$$ of $${\mathfrak {g}}\oplus {\mathfrak {h}}$$.
  In the text this bilinear the constituents are a sum over representations, $\varepsilon = \oplus _i \varepsilon _i$ where $\varepsilon _i\in ({{\varvec{R}}}_i, {{\overline{\varvec{r}}}}_i)$ and the trace projects onto the natural invariants. There are nonzero invariants as the trace derives from the $\mathrm{ad }_{{\mathfrak {e}}_8}$ which is real. For example, if $({{\varvec{R}}}_i,{{\overline{\varvec{r_i}}}})$ is complex representation, then the sum $\oplus _i$ contains both $({{\varvec{R}}}_i,{{\overline{\varvec{r_i}}}})$ and its conjugate representation $({{\overline{\varvec{R}}}}_i, {{\varvec{r_i}}})$, with the trace constructing the natural invariant.
  B.37
  $\,\text {Tr}\,_{\mathfrak {g}}$ and $\,\text {Tr}\,_{\mathfrak {h}}$ descend from the trace over ${\mathfrak {e}}_8$. They are understood to mean to contract the ${{\varvec{R}}}$ and ${{\varvec{r}}}$ indices in the appropriate way in order to get an invariant; if none exists, then the trace vanishes. We use (B.31) in the last two lines. We have written $\delta A_{\bar{\mu }}= \delta \Phi ^\Xi \delta _\Xi A_{\bar{\mu }}$ to represent a generalised variation of the ${\mathfrak {e}}_8$ gauge field. In the text this includes moduli and matter fields e.g. .

## C Some representation theory

Consider some Lie algebra ${\mathfrak {e}}$ and for this subsection only denote $a=1, \ldots ,\dim {\mathfrak {e}}$. We choose *A* to be anti-Hermitian $A^\dag = -A$. In terms of adjoint generators $T^a$:

$$\begin{aligned} A = A^a T^a, \qquad (A^a)^* = A^a, \qquad [T^a]^\dag = - T^a. \end{aligned}$$

Anti-Hermitian matrices for the fundamental and antifundamental satisfy

$$\begin{aligned}{}[T^a,T^b]=f^{abc}T^c, \quad [(T^*)^a,(T^*)^b]=f^{abc}(T^*)^c,\quad (f^{abc})^* = f^{abc}. \end{aligned}$$

We identify

$$\begin{aligned} T_{\overline{R}} = (T_R^a)^*. \end{aligned}$$

Let $\chi$ be in the fundamental and $\Psi$ in the antifundamental. The covariant derivatives are

$$\begin{aligned} \text {d}_A \chi = d \chi + A \chi , \qquad \text {d}_A \psi ^t = d\psi ^t + \psi ^t A. \end{aligned}$$

Decompose into type:

$$\begin{aligned} A = A_{0,1} + A_{1,0} = \left( A_{0,1}^a + A_{1,0}^a\right) T^a. \end{aligned}$$

For $A^a$ to be a real form, we require

C.1 $$\begin{aligned} \left( A^a_{0,1}\right) ^* = A_{1,0}^a, \qquad \left( A^a_{1,0}\right) ^* = A_{0,1}^a. \end{aligned}$$

This is consistent with $A_{0,1}^\dag = - A_{1,0}$ and $A_{1,0}^\dag = - A_{0,1}$.

As in the paper we define

$$\begin{aligned} A = \mathcal {A}- \mathcal {A}^\dag , \end{aligned}$$

where

$$\begin{aligned} \mathcal {A}:= A_{0,1}, \qquad \mathcal {A}^\dag := - A_{1,0}. \end{aligned}$$

Now define the components of $\mathcal {A}$ and $\mathcal {A}^\dag$:

C.2

The conjugation property of $\mathcal {A}^a$ is:

C.3 $$\begin{aligned} \begin{aligned} (\mathcal {A}^a)^* = \left( A_{0,1}^a\right) ^* = A_{1,0}^a = - (\mathcal {A}^\dag )^a. \end{aligned} \end{aligned}$$

## Footnotes

1 I would like to thank Xenia de la Ossa for explaining this choice of basis to me.

2 It is important to note that the derivation here and in [5], no assumption is made about expanding around the standard embedding. $\mathcal {E}$ is not related to the tangent bundle.

3 An important open question is, when are singlet couplings are generated by worldsheet instantons? At least for vacua derived from linear sigma models, there are arguments that suggest that after summing over all worldsheet instantons all the singlet couplings vanish [12, 13]. Here we assume the vacua is well defined with a large radius limit, and so all singlet couplings vanish.

4 It may be useful to define $\bar{\Psi }_\mu ^a$ given by $\bar{\Psi }_\mu ^a =( \Psi _{\bar{\mu }}^a)^*$ so that *a* is a real index and $\Phi _{1,0}^a = -\, \psi ^{*\,a} = -\, \bar{\Psi }^a_{1,0}$.

5 We do not consider the universal multiplet, the $d=4$ dilaton and *B*-field, which decouples.

6 The form of this integrand is due to Xenia de la Ossa who suggested to me in private conversation.

7 In the literature a different ansatz is proposed for the superpotential:  After careful calculation one can check , and so there are no ${{\varvec{1}}}, {{\varvec{1}}}^2, {{\varvec{1}}}^3$ couplings. To what extent this reproduces singlet couplings to higher order is an interesting question.

8 Many examples of relations involving complex structure do not hold for all $y_0\in \mathcal {M}$. A simple example is $\text {d}J$. Although for any fixed complex structure $\text {d}J|_{y=y_0} = 0$, differentiating we get something nonzero $\partial _\alpha \text {d}J = \partial \Delta _\alpha |_{y=y_0} \ne 0$.
